# Neutrophil serine protease 4 is required for mast cell-dependent vascular leakage

**DOI:** 10.1038/s42003-020-01407-0

**Published:** 2020-11-19

**Authors:** Andrew P. AhYoung, Sterling C. Eckard, Alvin Gogineni, Hongkang Xi, S. Jack Lin, Stefan Gerhardy, Christian Cox, Qui T. Phung, Jason A. Hackney, Anand Kumar Katakam, Mike Reichelt, Patrick Caplazi, Paolo Manzanillo, Juan Zhang, Merone Roose-Girma, Lucinda W. Tam, Robert J. Newman, Aditya Murthy, Robby M. Weimer, Jennie R. Lill, Wyne P. Lee, Michele Grimbaldeston, Daniel Kirchhofer, Menno van Lookeren Campagne

**Affiliations:** 1 Department of Early Discovery Biochemistry, 1 DNA Way, South San Francisco, CA 94080 USA; 2 Department of Immunology, 1 DNA Way, South San Francisco, CA 94080 USA; 3 Department of Biomedical Imaging, 1 DNA Way, South San Francisco, CA 94080 USA; 4 Department of Microchemistry, Proteomics, Lipidomics, 1 DNA Way, South San Francisco, CA 94080 USA; 5 Department of Bioinformatics, 1 DNA Way, South San Francisco, CA 94080 USA; 6 Department of Pathology, 1 DNA Way, South San Francisco, CA 94080 USA; 7 Department of Translational Immunology, 1 DNA Way, South San Francisco, CA 94080 USA; 8 Department of Molecular Biology, 1 DNA Way, South San Francisco, CA 94080 USA; 9 Department of Cancer Immunology, 1 DNA Way, South San Francisco, CA 94080 USA; 10 OMNI-Biomarker Development, Genentech Inc, 1 DNA Way, South San Francisco, CA 94080 USA; 11 Present Address: Department of Inflammation and Oncology, Amgen Research, Amgen, 1120 Veterans Boulevard, South San Francisco, CA 94080 USA

**Keywords:** Immunology, Rheumatology

## Abstract

Vascular leakage, or edema, is a serious complication of acute allergic reactions. Vascular leakage is triggered by the release of histamine and serotonin from granules within tissue-resident mast cells. Here, we show that expression of Neutrophil Serine Protease 4 (NSP4) during the early stages of mast cell development regulates mast cell-mediated vascular leakage. In myeloid precursors, the granulocyte–macrophage progenitors (GMPs), loss of NSP4 results in the decrease of cellular levels of histamine, serotonin and heparin/heparan sulfate. Mast cells that are derived from NSP4-deficient GMPs have abnormal secretory granule morphology and a sustained reduction in histamine and serotonin levels. Consequently, in passive cutaneous anaphylaxis and acute arthritis models, mast cell-mediated vascular leakage in the skin and joints is substantially reduced in NSP4-deficient mice. Our findings reveal that NSP4 is required for the proper storage of vasoactive amines in mast cell granules, which impacts mast cell-dependent vascular leakage in mouse models of immune complex-mediated diseases.

## Abstract (web summary)

AhYoung, Eckard et al. show that the expression of Neutrophil Serine Protease 4 (NSP4) during the early stages of mast cell development regulates the levels of histamine and serotonin in mast cell granules. This study reveals an important physiological function of NSP4 in mast cell-mediated vascular leakage in mice, establishing NSP4 as a potential therapeutic target for mast cell-dependent immune disorders.

## Introduction

Mast cells are tissue-resident immune cells that play a pivotal role in microbial defense, toxin neutralization, and tissue remodeling^1–4^. They contain electron-dense secretory granules that store inflammatory mediators^5^. Upon exposure to various stimuli including allergens that cross-link FcεRI, mast cells degranulate, rapidly releasing their granule contents, and triggering localized vascular leakage and tissue inflammation^6^. Stored mediators released upon antigen stimulation include the vasoactive amines histamine and serotonin, and mast cell proteases, such as tryptase, chymase, and carboxypeptidase A3^5^. Other secreted products, such as leukotrienes, and various chemokines and cytokines are de novo synthesized following mast cell activation and do not rely on sorting and packaging into granules^5^.

Mast cell stimuli include antibody–antigen immune complexes, activated complement, neuropeptides, and toxins. Immune complex formation is a critical step in adaptive immunity and results in the mobilization of both the innate and adaptive arms of the immune system to defend against invading pathogens and the toxins they release^7^. The immune complex-induced inflammatory response is characterized by changes in the microcirculation leading to impaired endothelial barrier function, plasma protein and fluid efflux, and extravasation of white blood cells. Histamine and serotonin release is the principal driver of mast cell-induced vascular permeability^8^. In addition, the release of the cytokine IL-1β by mast cells at sites of immune complex activation is critical for the recruitment of neutrophils^9^, which subsequently stimulates their own recruitment by releasing LTB4 and IL-1β^10^. While critical for the host immune response and tissue repair, vascular leakage induced by mast cells upon stimulation by antigen- or hapten-carrier immune complexes is also a key adverse reaction associated with a wide range of antigen-specific and idiopathic allergies^11^.

The formation and packaging of secretory granules with inflammatory mediators begin at the pro-granulocyte stage during granulocyte development^12,13^. In mast cells, the glycosaminoglycans (GAGs) heparin, heparan sulfate, or chondroitin sulfate, which are attached to the major mast cell proteoglycan serglycin, are essential for granule maturation and the retention of granule content^14–17^. Heparan sulfate and heparin polysaccharides are composed of sulfated disaccharide repeats, with heparin being sulfated to a larger extent compared to heparan sulfate^18,19^. In the low pH environment of secretory granules, the negatively charged heparin directly binds to positively charged histamine and serotonin^20,21^. Loss of heparin sulfation is associated with diminished levels of histamine, serotonin, and various mast cell proteins^14,15^. Thus, proper maturation of the granule is critical for proper mast cell effector functions.

Neutrophil serine proteases (NSPs) play critical roles in inflammation^22^ and in the neutrophil-mediated clearance of invading microbes^23^. The NSP family is a group of four homologous proteases that include neutrophil elastase (NE), cathepsin G (CG), proteinase 3 (PR3), and NSP4. NSPs are functionally redundant, with defects often only being revealed in mice deficient in at least two NSP members, such as NE/CG-deficient mice^24,25^, NE/PR3-deficient mice^26^, and dipeptidyl peptidase I-deficient mice, which lack all four NSPs^24^. Human NSP4 (encoded by the gene *PRSS57*) is a trypsin-fold protease stored in neutrophil azurophilic granules^27,28^. The gene *PRSS57* was originally identified by yeast signal trap and computational mining of human cDNA libraries^29^. NSP4-deficient mice (*Prss57−/−* mice) have been generated^30^, but no in vivo function has so far been described.

Three lines of evidence suggest that NSP4 could play roles that are distinct from other NSPs. First, NSP4 has been conserved for over 400 million years from bony fish to humans, predating the emergence of the other 3 NSP members^27,31^. Second, NSP4 is the only neutrophil protease that is capable of cleaving substrates after arginine residues^27,28,32^. Third, NSP4 possesses an enzyme active site that has the ability to process substrates with posttranslationally modified arginine residues, such as methylarginine and citrulline, that are typically uncleavable by other arginine-specific proteases^32^. These attributes suggest that NSP4 could have potential nonredundant roles in granulocyte function.

Herein, we demonstrate that NSP4 plays an essential role in mast cell biology. NSP4 is expressed during early mast cell development in bone marrow precursor cells and is critical for maintaining normal levels of histamine and serotonin in the secretory granules of the developing mast cells. NSP4-deficiency in mice leads to reduced levels of histamine and serotonin, resulting in reduced vascular leakage in models of passive cutaneous anaphylaxis (PCA) and acute arthritis. Given the high burden of angioedema in the general population^11,33^, this finding has implications for therapeutic targeting of immune-complex-induced allergic reactions.

## Results

### NSP4 is expressed in granulocyte–macrophage progenitors

**Fig. 1:**
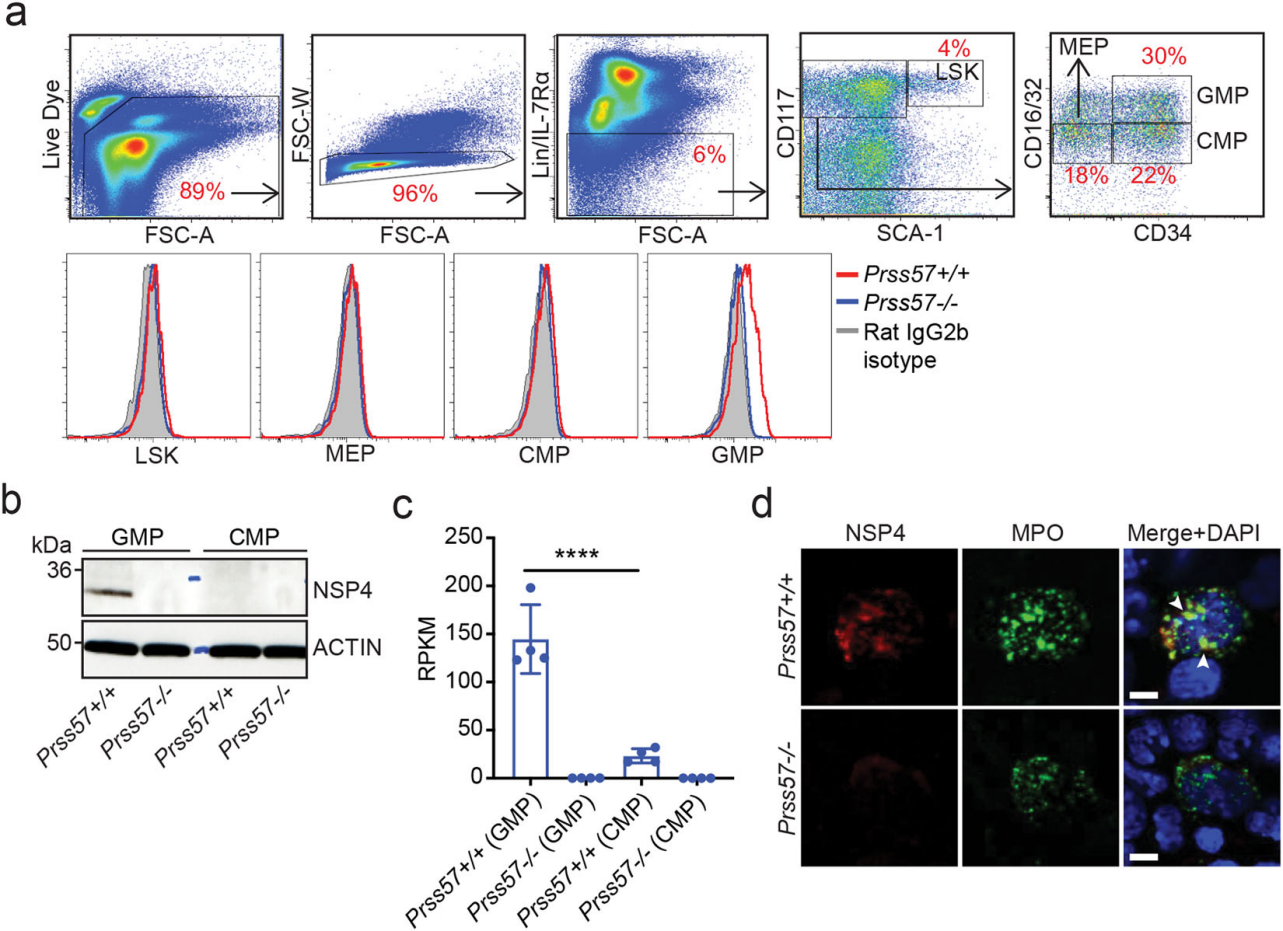
NSP4 is expressed in bone marrow-resident, granulocyte-macrophage progenitors (GMPs). Identification of the NSP4-expressing cell type. **a** Intracellular staining and flow cytometry analysis for NSP4 expression in bone marrow cells isolated from wild-type and *Prss57*−/− littermates. Density plots describe the gating strategy and histogram overlays of *Prss57*+/+, *Prss57*−/−, and rat IgG2b isotype staining are shown for each gated population: (1) LSK: Lin−, SCA-1+, CD117+ cells; (2) megakaryocyte/erythrocyte progenitors (MEP): Lin−, SCA-1−, CD117+, CD34−, CD16/32^lo^; (3) common myeloid progenitors (CMP): Lin−, SCA-1−, CD117+, CD34+, CD16/32^lo^; (4) granulocyte-macrophage progenitors (GMP): Lin−, SCA-1−, CD117+, CD34+, CD16/32^hi^. **b** Western blot for NSP4 in GMPs and CMPs. Data shown are representative of at least three independent experimental repeats. **c** *Prss57* expression by RNA sequencing, shown as reads per kilobase per million (RPKM). Data are presented as mean ± s.d.; *n* = 4 biological replicates per genotype; ****P < 0.0001; two-way ANOVA and Bonferroni post hoc test. **d** Immunofluorescence staining for NSP4, myeloperoxidase (MPO), and nucleus (DAPI counterstain) in a total bone marrow smear; NSP4 and MPO colocalization is indicated by arrowheads; scale bar, 5 μm.

To study the function of NSP4, we generated two strains of NSP4-deficient mice (*Prss57−/−*): strain 1 contains a deletion in exons 2 and 3^30^, and strain 2 contains a deletion in exon 2 only (Supplementary Fig. 1a). NSP4 deletion was confirmed at the mRNA and protein level by quantitative polymerase chain reaction (qPCR) and western blot of total bone marrow cells (Supplementary Fig. 1b). Neither strain exhibited any gross phenotypic or histological abnormalities^30^, nor alterations in immune cell populations of the blood, spleen, and bone marrow, except for a slight, but significant, increase in peripheral blood basophils and eosinophils (Supplementary Fig. 2a–c). NSP4 protein was expressed in bone marrow-resident neutrophils (Supplementary Fig. 3a, b) and bone marrow granulocyte precursors (Fig. 1), corroborating previous reports^27,34^. Further characterization of bone marrow precursors by intracellular flow cytometry (FACS)-staining, western blot, and qPCR revealed the specific expression of NSP4 in a subset of myeloid progenitors called granulocyte–macrophage progenitors (GMPs; defined as Lin−, SCA-1−, CD117+, CD34+, CD16/32^hi^) (Fig. 1a–c). In the bone marrow, NSP4 co-localized with myeloperoxidase (MPO) indicating its subcellular localization in azurophilic granules (Fig. 1d).

To investigate the role of NSP4 in GMPs, we performed bulk RNA sequencing on FAC-sorted wild-type and *Prss57−/−* GMPs (Supplementary Fig. 4a, b). There were no gross differences either at the global transcriptome level (Supplementary Fig. 4b and Supplementary Table 1) or in the expression (of RNA and protein) of selected mast-cell relevant genes comparing wild-type and *Prss57*−/− mice (Supplementary Fig. 4c, d). GMPs differentiate into various cells of the myeloid innate immune system, such as neutrophils and mast cells^35^. To investigate whether NSP4 expression in GMPs affects neutrophil function, we performed ex vivo assays using bone marrow-resident neutrophils isolated from wild-type and *Prss57−/−* mice. We found no effect of NSP4 deletion on neutrophil-mediated bacterial killing, neutrophil migration, neutrophil production of reactive oxygen species, and expression of neutrophil serine proteases and MPO (Supplementary Fig. 5a–d), suggesting that NSP4 does not contribute to these neutrophil effector functions.

### NSP4-deficient mast cells have altered secretory granules, and reduced histamine and serotonin levels

Bone marrow GMPs differentiate into circulating mast cell precursors (MCp), which home to nearly all vascularized tissues to complete their maturation into mucosal and connective tissue-resident mast cells^35^. To determine if NSP4 expression in GMPs regulates the homing potential of mast cells, we examined mast cell levels in various tissues. We found no effect of NSP4 deletion on the distribution of connective-tissue mast cells in the mesentery and mucosal mast cells in the gall bladder (Supplementary Fig. 6a, b). Consistent with these in vivo findings, we demonstrated in vitro that NSP4 deletion did not affect the differentiation of bone marrow-derived GMPs or peritoneum-derived progenitors into mature connective-tissue-like mast cells (BMMCs or PCMCs) under the influence of interleukin-3 (IL3) and stem cell factor (SCF) (Supplementary Fig. 6c)^36,37^. Taken together, our results suggest that NSP4 expression in GMPs is not necessary for mast cell homing and maturation.

**Fig. 2:**
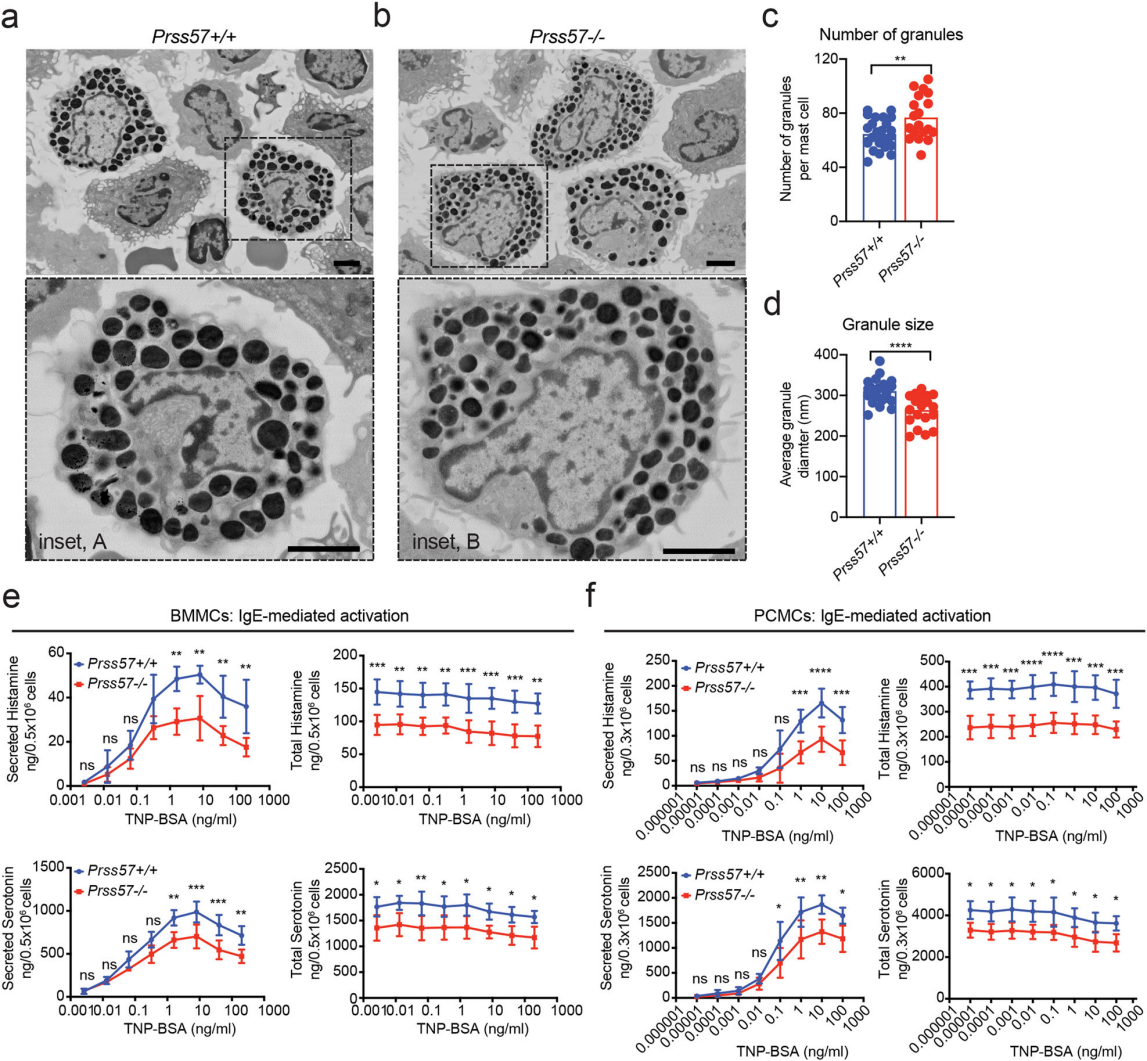
Mast cells obtained from NSP4-deficient mice have abnormal granule features and reduced levels of granule-stored histamine and serotonin. Ultrastructural analysis of primary peritoneal cell suspension by scanning electron microscopy (SEM). Representative scanning electron micrographs showing mast cells (dashed box) from *Prss57*+/+ (**a**) and *Prss57*−/− littermates; scale bar, 2 μm; inset scale bar, 2 μm. **b** High magnification views of *Prss57*+/+ (inset A) and *Prss57*−/− (inset B) mast cells. scale bar, 2 μm; inset scale bar, 2 μm. Quantification of the number (**c**) and size (**d**) of electron-dense secretory granules present in mast cells based on representative SEM micrographs. Data are presented as mean ± s.d., *n* = 26 *Prss57*+/+ mast cells, and *n* = 19 *Prss57*−/− mast cells were used for quantification; **P < 0.01, ****P < 0.0001; Student’s *t* test. Data shown are representative of at least two independent experimental repeats. **e**, **f** Comparing histamine and serotonin levels in mast cells derived from wild-type and *Prss57*−/− littermates and differentiated in vitro. Total or secreted levels of histamine and serotonin following IgE-mediated activation of BMMCs (**e**) and PCMCs (**f**). Data are depicted as mean ± s.d., *n* = 4 biological replicates per genotype; *P < 0.05, **P < 0.01, ***P < 0.001, ****P < 0.0001; two-way ANOVA and Bonferroni post hoc test.

To determine if NSP4 affects mast cell secretory granules, we performed scanning electron microscopy on freshly isolated peritoneal mast cells. We found an ~18% increase in the number of granules and a ~15% decrease in granule size in *Prss57−/−* mast cells (Fig. 2a–d). To determine whether the morphological abnormalities in mast cell granules is associated with changes in the levels of granule-stored mediators, we measured the release of various mast cell mediators upon stimulation with immunoglobulin E (IgE)- or IgG-containing immune complexes. NSP4 deletion resulted in a significant decrease in both the total and secreted levels of histamine (~35%) and serotonin (~25%) in either IgE-sensitized BMMCs (Fig. 2e) and PCMCs (Fig. 2f), or IgG-sensitized PCMCs (Supplementary Fig. 7a). The ratio of secreted histamine or serotonin to their total levels in mast cells was similar between wild-type and *Prss57−/−* cells (Supplementary Fig. 7b–d), indicating that loss of NSP4 did not affect mast cell degranulation function. While we have not determined how NSP4 loss changes granule morphology, it could be secondary to loss of histamine, as exemplified by the altered secretory granule morphology in mast cells lacking histidine decarboxylase (HDC)^38^.

**Fig. 3:**
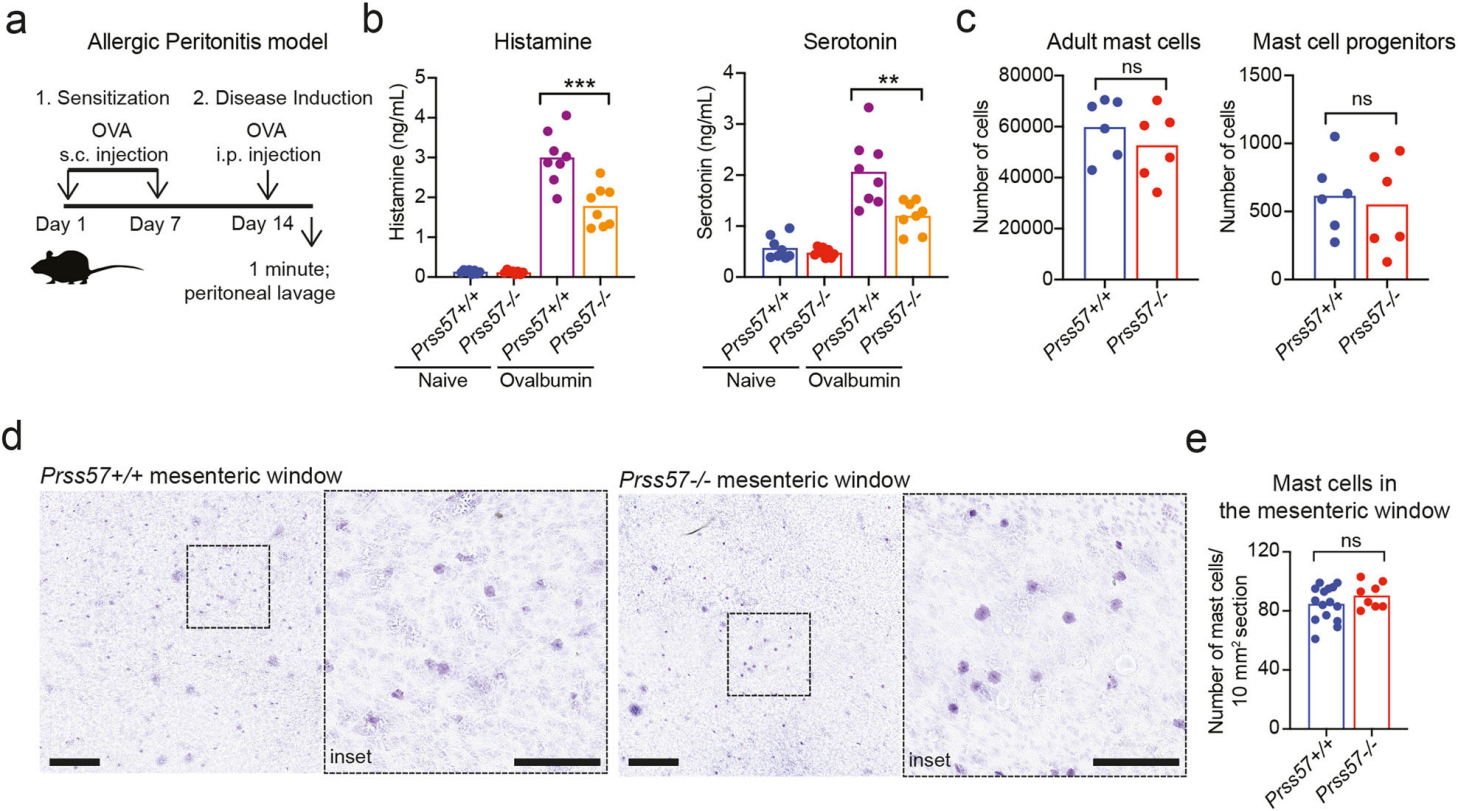
NSP4 deletion decreases histamine and serotonin but does not impact mast cell numbers in the peritoneum in vivo. **a** Experimental setup for ovalbumin (OVA)-induced allergic peritonitis mouse model. **b** Histamine and serotonin levels in the peritoneal cavity of wild-type and *Prss57*−/− littermates before and after OVA-induced allergic peritonitis. Bars represent mean; *n* = 8 biological replicates per genotype; **P < 0.01 and ***P < 0.001; two-way ANOVA and Bonferroni post hoc test. **c** Flow cytometry-based quantification of adult mast cells and mast cell progenitors isolated from the peritoneal cavity of *Prss57*+/+ and *Prss57*−/− littermates. Bars represent mean; *n* = 6 biological replicates per genotype; Student’s *t* test. **d** Toluidine blue-stained flat mounts of the mesenteric window from *Prss57*+/+ and *Prss57*−/− littermates. Scale bar, 0.5 mm; inset scale bar, 0.25 mm. **e** Mast cells were quantified in 10 mm^2^ regions of interest (ROIs) in Toluidine blue-stained mesenteric window preparations. Fifteen randomly selected ROIs from three *Prss57*+/+ mice, and eight randomly selected ROIs from three *Prss57*−/− littermates were quantified, ns (not significant), *P* > 0.05, Student’s *t* test. Data shown are representative of at least two independent experimental repeats.

To confirm the decrease of histamine and serotonin levels in vivo, we employed a model of allergic peritonitis (Fig. 3a). In the peritoneal lavage of *Prss57−/−* mice, we found a significant decrease in histamine (~40%) and serotonin (~42%) compared to wild-type mice (Fig. 3b). While both mast cells and basophils can contribute to histamine and serotonin release in the peritoneal cavity, mast cells outnumber basophils by over two orders of magnitude^39^, suggesting that the majority of histamine released in the peritoneal cavity is mast cell-derived. The number of mast cells in the peritoneal cavity and mast cells residing in the mesenteric window did not differ significantly between wild-type and *Prss57−/−* mice (Fig. 3c–e), indicating that the decrease in histamine and serotonin was not due to reduced numbers of *Prss57−/−* mast cells, but to reduced amounts of histamine and serotonin stored in their granules

To investigate whether NSP4 affects the levels of other proinflammatory mediators, we quantified the total and secreted levels of other proinflammatory factors by enzyme-linked immunosorbent assay (ELISA). Using BMMCs, we found no effect of NSP4 deletion on the levels of leukotriene-B_4_ (LTB_4_), TNF-α, IL-6, IL-13, C–C motif chemokine ligand 2 (CCL2), and macrophage inflammatory protein 1 alpha (MIP-1α) (Supplementary Fig. 8). Consistent with these findings, bulk RNA sequencing revealed no gross differences in the transcriptome of wild-type and *Prss57−/−* BMMCs (Supplementary Fig. 9a). These results were confirmed by measuring the protein expression of mast cell proteases, such as mast cell tryptase (MCPT6), chymase (CMA1), and carboxypeptidase 3 (CPA3) (Supplementary Fig. 9b). We further verified these findings using FAC-sorted primary peritoneal mast cells from wild-type and *Prss57−/−* mice, demonstrating comparable protein expression of MCPT6 and CPA3 (Supplementary Fig. 9c, d).

### Mast cell histamine and serotonin are regulated by NSP4 expression in GMPs at the early stage of mast cell maturation

**Fig. 4:**
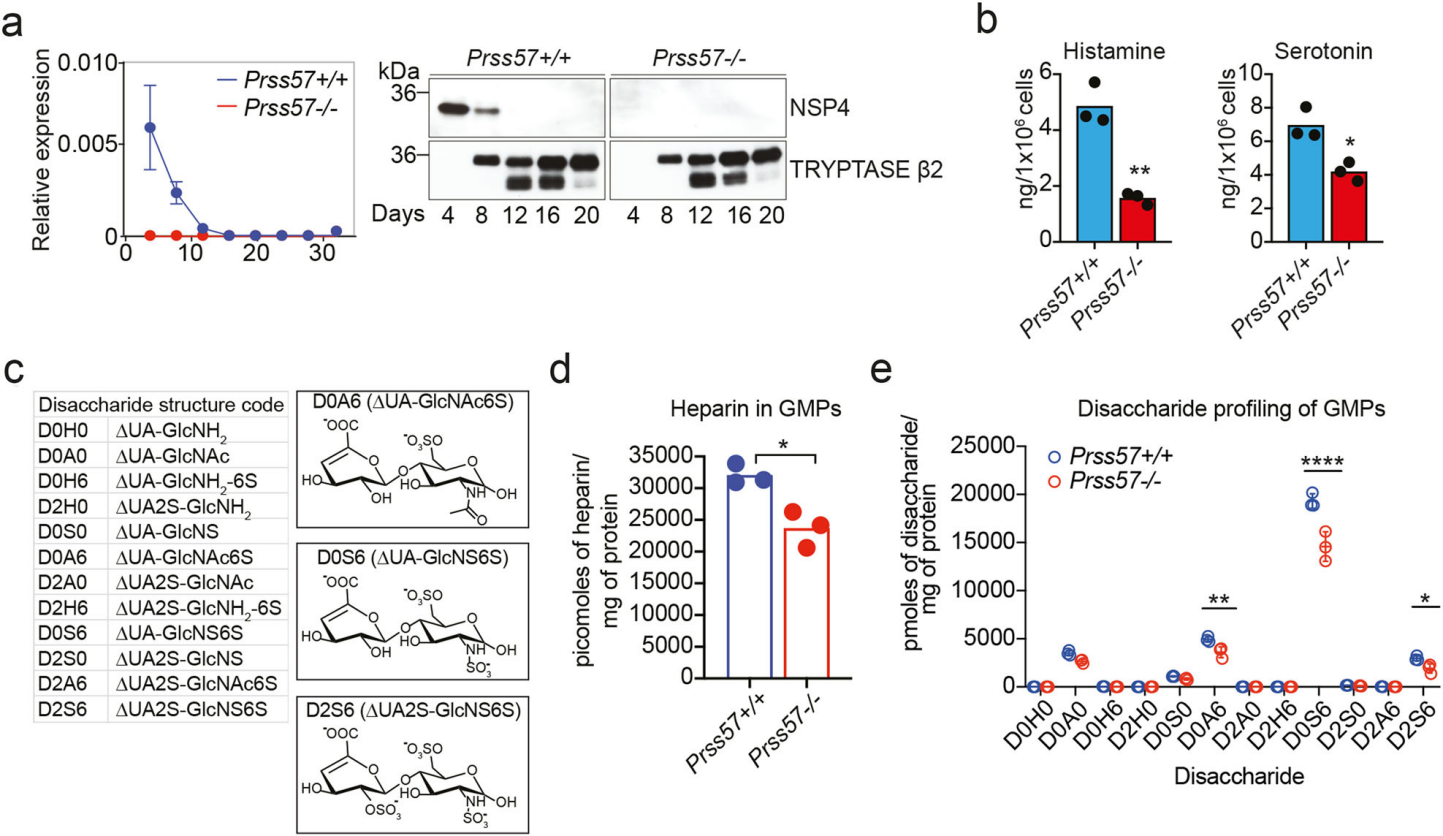
NSP4 regulates histamine, serotonin, and heparin/heparan sulfate levels in GMPs. **a** NSP4 expression monitored by qPCR and Western blot during the differentiation of GMPs to mast cells in vitro. **b** Histamine and serotonin levels in “mast cell-primed” GMPs of *Prss57*+/+ and *Prss57*−/− littermates. Bars represent mean; *n* = 3 biological replicates per genotype; *P < 0.05, **P < 0.01; Student’s *t* test. **c** Disaccharide structure code (DSC) for the representation of all disaccharides derived from any glycosaminoglycan. Chemical structure of the sulfated disaccharides that are enriched in GMP and BMMC samples. **d**, **e** Total levels of heparin/heparan sulfate and its corresponding disaccharide profile in GMPs. Data are presented as mean (**b**, **d**) or mean ± s.d. (**c**, **e**); *n* = 3 or 4 biological replicates per genotype; *P < 0.01, **P < 0.001, ***P < 0.0001 for indicated comparisons; statistical significance was determined by the unpaired Student’s *t* test corrected for multiple comparisons using the Holm–Sidak method. Data shown are representative of at least two independent experimental repeats.

To understand how NSP4 regulates histamine and serotonin in mast cells, we examined NSP4 expression by western blot and qPCR during the differentiation of FACS-sorted GMPs to BMMCs ex vivo (Supplementary Fig. 10). We observed a substantial decrease in *Prss57* mRNA and NSP4 protein expression by day 8, and a complete loss by day 12 of differentiation (Fig. 4a). On day 12, more than 99% of the cells in culture were fully differentiated mast cells based on their expression of c-KIT and FcεR1α (Supplementary Fig. 10). This is consistent with the increase in tryptase protein, which appears upon the induction of mast cell maturation and coincides with the loss of NSP4 expression (Fig. 4a).

The finding that NSP4 deficiency resulted in reduced histamine and serotonin levels in mature mast cells suggested that the storage of these vasoactive amines is regulated by NSP4 expressed in GMPs during the early stages of mast cell development. To address this hypothesis, we quantified histamine and serotonin levels in GMPs using mass spectrometry. Given the limited number of GMPs in the mouse bone marrow, FACS-sorted GMPs were expanded in mast cell differentiation culture media for 4 days prior to analysis. Flow cytometry analysis of these “mast cell-primed GMPs” after 4 days in culture showed that only a small percentage (~2–2.5%) have differentiated to mast cells (cKIT+, FcεR1α+), whereas the majority are immature-like progenitors (Supplementary Fig. 10). Mass spectrometry analysis of these mast-cell-primed GMPs showed a significant decrease in histamine (~70%) and serotonin (~30%) in *Prss57−/−* GMPs compared to wild-type control (Fig. 4b). Bulk RNA sequencing and western blot analyses of primary GMPs (Supplementary Fig. 4) showed no effect of NSP4 deletion on expression of proteins involved in histamine biosynthesis (HDC) or transport into secretory granules (vesicular amine transporter 2, SLC18A2 or VMAT2) (Supplementary Fig. 4d) suggesting that the decrease in histamine and serotonin is not associated with their biosynthesis or transport. Consistent with these findings in GMPs, we found similar protein expression levels of HDC and VMAT2 in primary wild-type and *Prss57−/−* peritoneal mast cells (Supplementary Fig. 9d).

Granule maturation and the storage of preformed mediators are tightly regulated by many factors, including heparin GAGs, which are covalently attached to the core protein serglycin (SRGN)^14,15,17^. In the acidic environment of mast cell granules, histamine is present in the protonated form, favoring direct electrostatic interactions with the highly negatively charged heparin^14,15,21^. This interaction is essential for histamine and serotonin storage, as genetic disruption of either SRGN or N-deacetylase/N-sulfotransferase (*Ndst2*), the enzyme responsible for heparin sulfation, resulted in diminished levels of histamine and serotonin^14,15^. We confirmed by western blot that SRGN and NDST2 expression in GMPs and BMMCs were not affected by NSP4 deletion (Supplementary Fig. 4d and Supplementary Fig. 9d). To determine whether NSP4 deletion affects the levels of heparin/heparan sulfate GAGs, we analyzed primary wild-type and *Prss57−/−* GMPs using an MS-based approach (glycan reductive isotope labeling combined with liquid chromatography/mass spectrometry (GRIL–LC/MS)), which enabled qualitative and quantitative analysis of heparin/heparan sulfate GAGs (Fig. 4c)^19,40^. We observed a ~35% reduction in the total amount of heparin/heparan sulfate GAGs in *Prss57−/−* GMPs compared with wild-type GMPs (Fig. 4d). The reduction is consistent with an overall decrease in three disaccharide species: (a) the mono-sulfated ΔUA-GlcNAc(6S) (denoted D0A6; ~27% reduction); (b) the di-sulfated ΔUA-GlcNS(6S) (D0S6; ~25% reduction); and (c) the tri-sulfated ΔUA(2S)-GlcNS(6S) (D2S6; ~35% reduction) (Fig. 4e). We note that the di-sulfated species (ΔUA-GlcNS(6S)) dominates over the tri-sulfated disaccharides (ΔUA(2S)-GlcNS(6S)), suggesting that heparan sulfate is the prevailing GAG in the mast cell-primed GMPs. We hypothesize that the overall reduction in heparin/heparan sulfate GAGs in *Prss57−/−* GMPs might contribute to the reduction in histamine and serotonin through altered storage capacity in granules. Additional studies are required to elucidate the link between by NSP4 expression and the levels of histamine, serotonin, and heparin/heparan sulfate GAGs in GMPs.

### NSP4 is required for mast cell-mediated vascular leakage in skin and joints

**Fig. 5:**
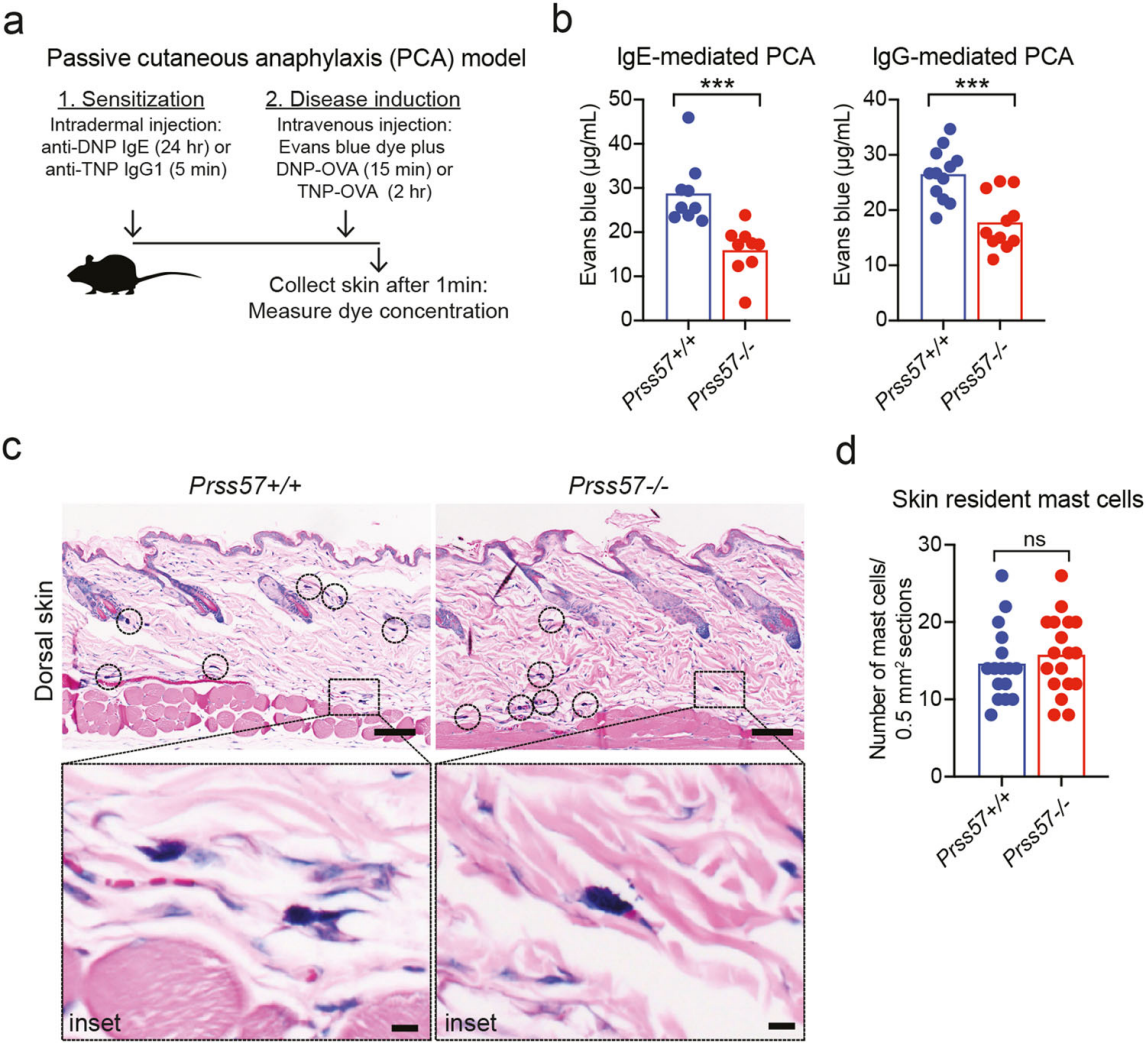
NSP4 is required for immune complex-induced vascular leakage in the skin. **a** Experimental setup for the passive cutaneous anaphylaxis (PCA) mouse model. **b** Vascular leakage in the skin of *Prss57*−/− mice and *Prss57*+/+ littermates based on extravasation of Evans blue dye in the IgE-mediated or IgG-mediated PCA model described in (**a**). Bars represent mean; *n* = 9–12 biological replicates per genotype; ***P < 0.001; Student’s *t* test. **c** Giemsa-stained cross-section of haired-skin (dorsum) isolated from *Prss57*−/− mice and wild-type littermates. The mast cells residing in the skin are connective-tissue mast cells, which are mostly located in the deep dermis. Scale bar, 100 μm; inset scale bar, 10 μm. **d** Mast cells were quantified in randomly selected 0.5 mm^2^ regions of interest (ROIs) in the section of Giemsa-stained tissues obtained from *Prss57*+/+ and *Prss57*−/− mice. Bars represent mean; 16 randomly selected ROIs in 9 sections from 2 *Prss57*+/+ littermates, and 18 randomly selected ROIs in nine sections from two *Prss57*−/− mice were quantified; Student’s *t* test. Data shown are representative of at least two independent experimental repeats.

Mast cell activation contributes to the pathogenesis of allergic and autoimmune diseases. The release of histamine by activated mast cells has been shown to stimulate vasodilation and tissue vascular permeability^6,8,41,42^. To assess the consequences of reduced histamine and serotonin levels in *Prss57−/−* mast cells, we used a mast cell-dependent model of IgE- or IgG-mediated Passive Cutaneous Anaphylaxis (PCA) (Fig. 5a)^43,44^. Compared to wild-type mice, vascular permeability in the skin of *Prss57−/−* mice was substantially diminished upon IgE stimulation (by >45%) and IgG stimulation (by 32%) (Fig. 5b). Histological analyses of wild-type or *Prss57−/−* skin revealed no change in the number of skin-resident mast cells (Fig. 5c, d), suggesting that the decrease in vascular leakage was likely due to reduced mast cell histamine and serotonin levels.

**Fig. 6:**
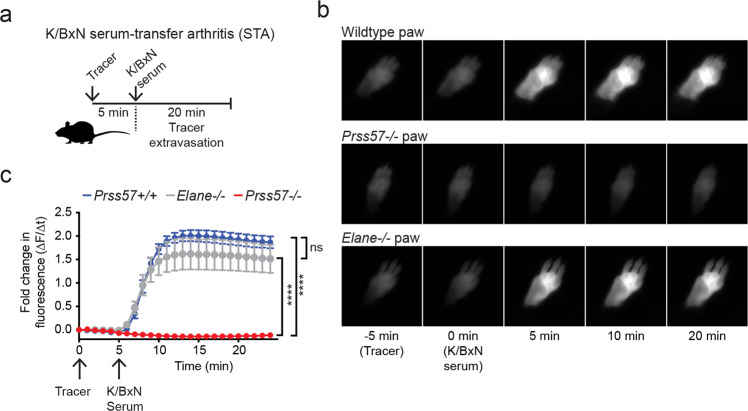
NSP4 is required for immune complex-induced vascular leakage in the joints. **a** Experimental setup for the K/BxN serum transfer arthritis (STA) mouse model. Representative time-lapse images (**b**) and quantification (**c**) of vascular leakage in the paws of *Prss57*+/+ and *Prss57*−/− littermates and neutrophil elastase-deficient (*Elane*−/−) mice. Data are presented as mean ± s.d.; *n* = 4 biological replicates per genotype; ****P < 0.0001; two-way ANOVA and Bonferroni post hoc test. Data shown are representative of at least three independent experimental repeats.

To determine if vascular leakage is impaired in other tissues, we examined the joint microvasculature using a mouse model of immune complex-mediated arthritis (K/BxN serum transfer arthritis (STA), Fig. 6a). In this model, serum autoantibodies against the ubiquitously expressed antigen glucose-6-phosphate isomerase elicit rapid, joint-restricted vascular permeability, which is later accompanied by joint pain, swelling, cartilage loss, and bone erosion^45^. Acute vascular permeability is driven in parallel by immune complex-induced mast cell activation and histamine release, and by FcγR-mediated activation of neutrophils^8^. As expected, the transfer of K/BxN arthritic serum into wild-type mice rapidly induced joint permeability (Fig. 6b, c). In contrast, joint vascular leakage was completely abolished in *Prss57−/−* mice. This phenotype was specific to NSP4 deletion as mice that lacked neutrophil elastase (*Elane−/−*), a related neutrophil serine protease, still developed joint vascular leakage (Fig. 6b, c). The complete loss of joint vascular permeability in the K/BxN serum transfer model contrasts with a partial (~40%) loss in serotonin and histamine when measured in peritoneal cavity-derived mast cells in vitro. Hence, it is possible that NSP4 regulates processes in addition to histamine and serotonin storage in secretory granules of mast cells that could contribute to the phenotype observed in the K/BxN model.

**Fig. 7:**
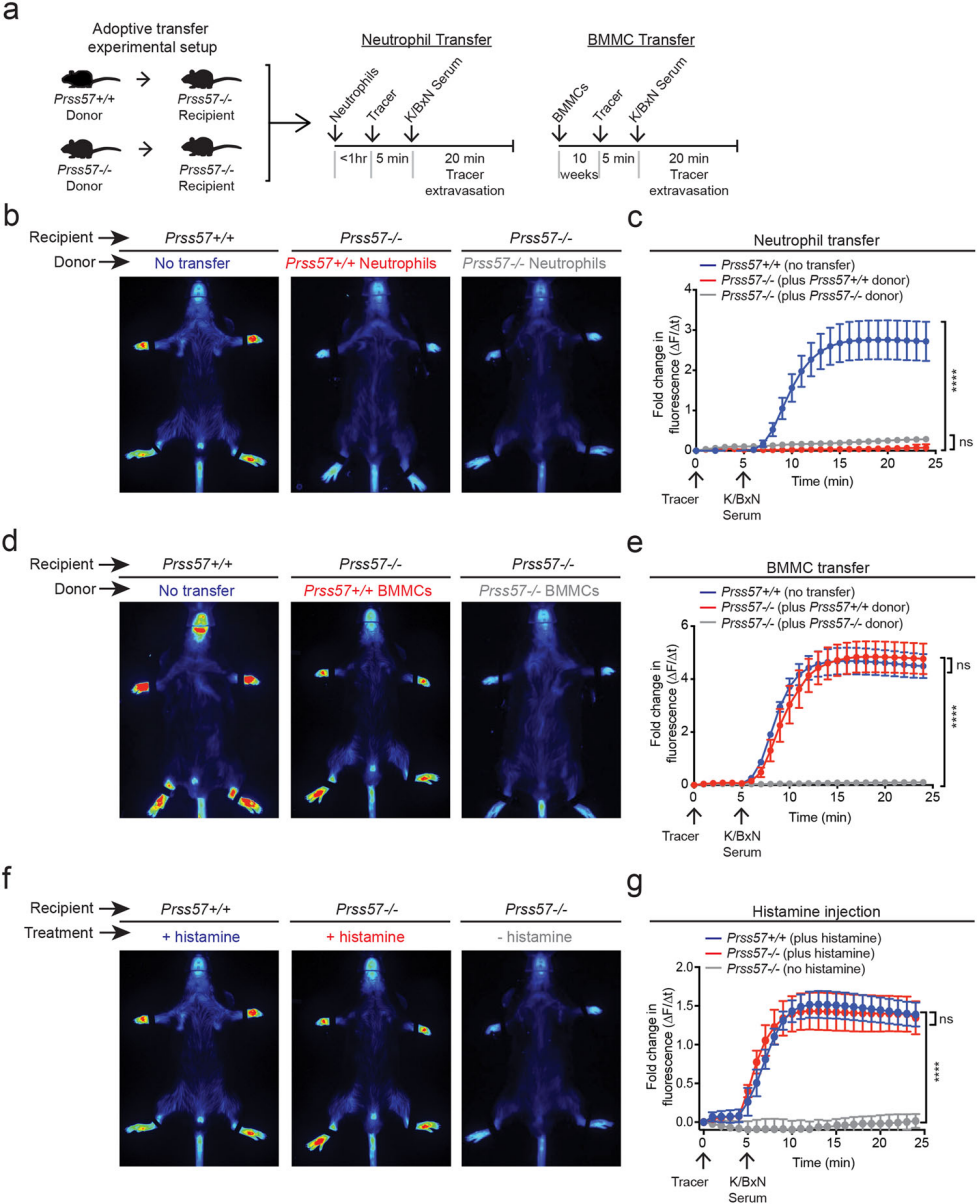
Mast cells and histamine are required for NSP4-dependent, immune complex-induced vascular leakage. **a** Experimental setup for the adoptive transfer of neutrophils and mast cells in the K/BxN STA model. Neutrophils and BMMCs were isolated from *Prss57*+/+ and *Prss57*−/− littermates. **b** Representative images and **c** quantification of vascular leakage following neutrophil transfer and K/BxN serum. Data are presented as mean ± s.d.; *n* = 5 biological replicates per genotype; ****P < 0.0001; two-way ANOVA and Bonferroni post hoc test. **d** Representative images and **e** quantification of vascular leakage following mast cell transfer and K/BxN serum injection into *Prss57*+/+ and *Prss57*−/− littermates. Data are presented as mean ± s.d.; *n* = 3–5 biological replicates per genotype; ****P < 0.0001; two-way ANOVA and Bonferroni post hoc test. **f** Representative images and **g** quantification of vascular leakage following histamine injection. Data are presented as mean ± s.d.; *n* = 3 biological replicates per genotype; ****P < 0.0001; two-way ANOVA and Bonferroni post hoc test. Data shown are representative of at least two independent experimental repeats.

We performed adoptive transfer experiments with neutrophils and mast cells to determine whether the protective phenotype in NSP4-knockout mice is associated with one or both cell types (Fig. 7a). The transfer of wild-type neutrophils into *Prss57−/−* mice showed no enhancement of joint leakage (Fig. 7b, c). In striking contrast, the transfer of wildtype but not *Prss57−/−* BMMCs completely restored vascular leakage to normal levels in *Prss57−/−* mice (Fig. 7d, e). To examine whether wild-type and *Prss57−/−* donor mast cells are equally capable of repopulating mast cell-replete mice, we used congenic *Cd45.1* mice as the recipients of the *Cd45.2* donor mast cells from *Prss57−/−* and *Prss57*+/+ mice. The differential expression of the *Cd45.1* or *Cd45.2* cell surface markers in recipient or donor mast cells, respectively, enabled us to monitor their grafting efficiency by FACS using allele-specific antibodies. Given the dispersion of intravenously grafted mast cells and the difficulty in tracking them, we used the peritoneal cavity as the site of localized mast cell engraftment. After 8 weeks post-transfer, we observed similar levels of wild-type and *Prss57−/−* BMMCs in the host peritoneum, indicating that NSP4 deletion does not affect mast cell grafting (Supplementary Fig. 11a, b). Notably, we found a substantial reduction (~80%) in the number of *Cd45.1*^*+*^ host mast cells in recipient mice following the transfer of *Cd45.2*^*+*^ donor-mast cells, suggesting that tissue-resident mast cells can be replaced by donor mast cells (Supplementary Fig. 11c). Donor wild-type or *Prss57*−/− PCMCs also displayed similar homing efficiency (Supplementary Fig. 11d, e). Finally, the systemic delivery of exogenous histamine was sufficient to overcome the absence of joint-restricted vascular permeability in *Prss57−/−* mice (Fig. 7f, g), indicating a critical role for systemic histamine in inducing joint-restricted vascular leakage. Collectively, our in vivo data demonstrate an essential role of NSP4 in regulating skin and joint vascular leakage in a mast cell- and histamine-dependent manner.

## Discussion

Although many aspects of acute vascular leakage and edema in allergic reactions and autoimmune disorders remain obscure, there is considerable evidence that mast cells and their granule-stored mediators histamine and serotonin contribute to disease pathogenesis. Herein, we identified NSP4 as a central modulator of vascular leakage in mouse models of PCA and acute arthritis. Intriguingly, we found that NSP4 is not expressed in differentiated mast cells, but in a specific subset of myeloid progenitors, the GMPs, which give rise to mast cells, suggesting an important role of NSP4 in mast cell development and mast cell-mediated inflammation. GMPs can also differentiate into neutrophils, which store NSP4 in their granules, as originally discovered by Perera et al.^27^. However, NSP4 deficiency did not cause any gross abnormalities in neutrophil effector function ex vivo, nor did neutrophils appreciably contribute to the vascular leakage in our K/BxN model, suggesting that the herein observed NSP4 function is specifically associated with the mast cell lineage.

Our results indicate that NSP4 neither regulates the differentiation of GMPs to mast cells nor affects mast cell trafficking to tissues. In addition, NSP4 does not regulate mast cell degranulation, or expression of proinflammatory cytokines, chemokines, and proteases. However, loss of NSP4 significantly reduced histamine and serotonin levels in GMPs and mast cells, lowered heparin/heparan sulfate levels in GMPs, and altered the size and quantity of secretory granules in mast cells. Based on these findings, one would expect a general defect in the granule storage of mast cell mediators. Yet, based on our extensive characterization by ELISA, western blot, and RNA sequencing, NSP4 deletion seems to specifically alter the levels of histamine and serotonin, but not other granule-stored mediators, such as mast cell tryptase, chymase, and carboxypeptidase A3 that are expressed in mature mast cells. This raises the possibility that NSP4 impacts only those granules that are generated in the early stages of mast cell differentiation when NSP4 is expressed. In support of this notion, we found that NSP4 and tryptase expression do not overlap; whereas NSP4 is highly expressed in GMPs and downregulated upon mast cell differentiation, tryptase is expressed only in the later stages of mast cell maturation. Thus, NSP4 might only regulate the granules that package histamine and serotonin, which concomitantly express NSP4 in GMPs during the early phase of mast cell development, but not the granules that are generated later^46–48^, resulting in a heterogenous granule population. Granule heterogeneity has been explained by the “targeting by timing” hypothesis, which proposes that granule proteins that are made concomitantly will end up in the same granule^12^. This view is consistent with the identification of functionally distinct granule subsets containing different cargo in mast cells^49^, and also applies to the heterogeneity of granules in neutrophils^50^. The “targeting by timing” concept may also explain the differential effect of NSP4 deletion on the levels of histamine and serotonin versus tryptase or other mast cell mediators. We cannot exclude the possibility that loss of NSP4 may affect other mast cell mediators that are expressed in GMPs.

We speculate that the observed changes in granule morphology and numbers in *Prss57*−/− mast cells could be secondary to their combined reduction in histamine, serotonin, and heparin/heparan sulfate GAGs. Previous studies have demonstrated that mast cells lacking HDC or heparin exhibit altered secretory granule morphology^14,38^. Further studies are required to determine the molecular mechanism by which NSP4 regulates secretory granule properties.

GMPs undergo substantial transformations, both transcriptionally and morphologically, during their differentiation into mast cells. We identified NSP4 as a granule-stored protein that controls several facets of secretory granule biology in developing mast cells, including the storage of vasoactive amines. The specific expression of NSP4 in GMPs suggests that early developmental processes in GMPs have a lasting effect on granule homeostasis, which is manifested in fully differentiated mast cells. Thus, NSP4 ensures that the developing mast cell acquires optimal levels of histamine and serotonin that are necessary to mount a robust inflammatory response to stimuli. The discovery that NSP4 deficiency affords significant protection against mast cell/histamine-dependent vascular leakage opens opportunities for therapeutic intervention of mast cell-dependent allergic and autoimmune diseases.

## Methods

### Generation of *Prss57−/−* mice

The Genentech institutional animal care and use committee responsible for ethical compliance approved all animal protocols. *Prss57−/−* mice (Strain 1) were previously described^30^. Strain 2 was generated by deleting exon 2 using the targeting construct depicted in Fig. 1a^51^. In the resulting vector, the 2412 base pair (bp) 5′ homology arm corresponds to GRCm38/mm10 chr10: 79,789,191–79,791,601 and the 2569 bp 3′ homology arm corresponds to chr10:79,786,365–79,788,689. The 501 bp region flanked by loxP sites (exon 2) corresponds to chr10:79,788,690–79,789,190. The final vector was confirmed by DNA sequencing, linearized, and used to target C2 (C57BL/6N) embryonic stem (ES) cells using standard methods (G418 positive and ganciclovir negative selection)^52^. Positive clones were identified using long-range PCR followed by sequence confirmation. Correctly targeted ES cells were subjected to karyotyping. Euploid gene-targeted ES cell clones were treated with Adeno-Cre to remove the floxed exon and ES cell clones were tested to identify clones with no copies of the PGK neomycin cassette and the correct sequence of the targeted allele was verified. The presence of the Y chromosome was verified before microinjection into albino BALB/c embryos. Germline transmission was obtained after crossing the resulting chimeras with C57BL/6N females. Genomic DNA from pups born was screened by long-range PCR to verify the desired gene-targeted structure before mouse colony expansion. For genotyping, the following primers were used: set 1 (5′-CAG ACA GAT AAG CAG AGT TGG-3′ and 5′-CCT TGT ACC TTC CTA CTA GAT TATC-3′) and set 2 (5′-CAG ACA GAT AAG CAG AGT TGG-3′ and 5′-ATG ATT GTT GGA ACC GAC TG-3′).

NSP4 deletion was confirmed by quantitative PCR and western blot using *Prss57−/−* bone marrow cells. *Elane*^*−/*^^−^ mice (strain B6.129×1-Elanetm1Sds/J) were obtained from The Jackson Laboratory. Age-matched wild-type and *Prss57−/−* littermates were used in all experiments.

### Generation of monoclonal antibodies to NSP4

Monoclonal antibodies were generated by immunizing *Prss57−/−* mice with recombinant mouse NSP4 in the presence of complete Freud’s adjuvant followed by fusion of the B-cell with a myeloma cell line^53^. Hybridoma clones were selected using an ELISA format to determine binding specificity to NSP4 but not other NSPs, granzyme M and complement factor D. Specificity was also assessed by immunohistochemistry on formalin-fixed, paraffin-embedded cell pellets (e.g., primary bone marrow cells and NSP4-transfected HEK293), western blotting on total bone marrow lysates, and binding studies with recombinant NSP4. Clones that fulfilled all screening criteria were reformatted into a rat IgG2b backbone for downstream assays.

### Intracellular staining coupled with flow cytometry

The identification of NSP4-expressing cells was done using flow cytometry. Rat anti-mouse NSP4 antibody (clone 29E7) and rat IgG2b isotype control were conjugated with AlexaFluor 488 (Abcam). Tissues were isolated from 2- to 3-month-old mice (i.e., bone marrow cells were harvested from femurs and tibiae, and blood was harvested by cardiac puncture). Red blood cells (RBC) were lysed with ammonium–chloride–potassium lysis buffer (Thermo Fisher Scientific). Remaining cells were washed and resuspended in FACS buffer (PBS, 0.5% bovine serum albumin (BSA), 2 mM EDTA, pH 8.0). Cells were incubated with the fixable yellow dead cell stain kit (Invitrogen) for 30 min on ice to exclude dead cells.

To stain bone marrow progenitors, cells were incubated with PerCP-Cy5.5-conjugated lineage antibody cocktail against CD2 clone RM2-5 (Biolegend), CD3 (145-2C11), CD4 (RM4-5), CD8 (53-6.7), CD19 (1D3), B220 (RA3-6B2), CD11b (M1/70), Gr-1 (RB6-8C5), and Ter119 (Ter119) (all from BD Biosciences) combined with PerCP-Cy5.5-α-IL-7Rα (A7R34), AlexaFluor 700-α-CD34 (RAM34) (both from eBiosciences), PE-α-CD16/32 (2.4G2), PE-Cy7-α-Sca-1 (D7), and APC-α-CD117 (2B8) (all from BD Biosciences).

To stain for bone marrow neutrophils, the cells were stained on ice for 30 min with AlexaFluor 700-α-Ly6G clone 1A8 (BD Biosciences) and AlexaFluor 647-α-Ly6B.2 clone 7/4 (Bio-Rad). For blood leukocytes, the cells were stained on ice for 30 minutes with PE-α-Ly6C (AL-21), AlexaFluor 700-α-Ly6G, PE-Cy7-α-CD11b (M1/70), PerCP-Cy5.5-α-CD45 (30-F11), PE-α-SiglecF (E50-2440) (all from BD Biosciences), PE-Cy7-α-CD3 (145-2C11), AlexaFluor 647-α-CD115, and eFluor 450-α-CD19 (1D3) (all from eBiosciences).

Intracellular staining for NSP4 was performed using the Fix & Perm Cell Permeabilization kit (Invitrogen) according to the manufacturer’s instruction. Stained cells were fixed with 100 μl of the fixation medium at room temperature for 15 min, followed by washing with PBS. The cells were resuspended in 100 μl of the permeabilization medium containing 5 μg/ml of AlexaFluor 488-α-NSP4 or a rat IgG2b isotype control antibody and incubated at room temperature for 30 min. After washing, the cells were resuspended in FACS buffer and analyzed on the LSR Fortessa flow cytometer. The data were acquired with BD FACS Diva software and analyzed with FlowJo software (GraphPad).

### Quantification of leukocyte populations by flow cytometry

Quantification of leukocyte populations was performed by flow cytometry. After RBC lysis, cells were stained in FACS buffer with the following fluorescent dye-conjugated antibodies: CD11b, CD3, CD19, Ly6G, SiglecF, Ly6B.2, CD49b (DX5), and FcεRI (MAR-1) (eBiosciences). A single-cell suspension of splenocytes was generated by homogenizing the spleen in the RPMI medium between frosted glass slides followed by filtering through a 70 μM filter. Splenocytes were stained with the following fluorescent dye-conjugated Antibodies, CD45, CD11b, Ly6G, Ly6B.2, CD19, CD4, CD8, SiglecF, F4/80 clone RM8 (eBiosciences), CD11c (HL3) and CCR3 (83103) (both from BD Biosciences). Peritoneal cells were harvested by peritoneal lavage with 5 ml of PBS containing 3% fetal bovine serum (FBS). Cells were stained with the following fluorescent dye-conjugated antibodies, CD11b, Ly6G, Ly6B.2, CD117, FcεRI, SiglecF, B220, F4/80, and TCRβ clone H57-597 (eBiosciences). Mast cell progenitors (MCp) in the peritoneum were identified by flow cytometry according to a previously described method^54^. Briefly, peritoneal cells were stained with PE-conjugated Antibodies against lineage markers B220, CD3, CD4, CD8, CD19, CD11b, Gr-1 and Ter119 in combination with PE-Cy7-α-CD117 clone 2B8 (BD Biosciences), FITC-α-integrin β (FIB504), AlexaFluor 700-α-CD16/32 (93), eFluor 450-α-FcεRI (MAR-1) (all from eBiosciences) and Biotin-α-ST2 clone DJ-8 (MD Biosciences) followed by APC-Streptavidin (BD Biosciences). Bone marrow progenitors were also stained and quantified as described above. All cells were analyzed on the LSR Fortessa flow cytometer. Total blood leukocytes, splenocytes, peritoneal leukocytes, and bone marrow progenitors were quantified by mixing an aliquot of single-cell suspension 1:1 with a known concentration of 6 μm Fluoresbrite YG microspheres (Polysciences) followed by counting by flow cytometry.

### In vitro differentiation of BMMCs and PCMCs

Bone marrow-derived mast cells (BMMCs) were generated as previously described^55^ with minor modifications. Lineage negative cells were enriched by lineage depletion of total bone marrow cells, and further FACS-sorted to isolate a pure population of GMPs (see Supplementary Fig. 4). GMPs were immediately cultured in mast cell differentiation media (containing RPMI 1640, 10% fetal bovine serum, 4 mM l-glutamine, 10 mM HEPES pH 7.5, 1% nonessential amino acids, 1 mM sodium pyruvate, 1% pen/strep, 50 μM β-mercaptoethanol, 25 ng/ml recombinant murine IL-3, and 25 ng/ml recombinant murine SCF; IL-3 and SCF were purchased from Peprotech) in a humidified 37 °C, 5% CO_2_ incubator. Cells were passaged every 4 days, and cell differentiation was monitored routinely by flow cytometry analysis for the expression of CD117 and FcεRIa surface receptors. Peritoneal cavity-derived mast cells (PCMCs) were generated as previously described^37^ with minor modifications. Briefly, peritoneal cells were collected by peritoneal lavage with 5 ml of PBS containing 3% FBS and seeded at 1 × 10^6^ cells/ml in mast cell differentiation media. Suspension cells were harvested and passaged every 4 days. After 3–4 weeks, more than 98% of the cells expressed CD117 and FcεRIa. BMMCs and PCMCs were used various in vitro assays.

### Mast cell degranulation assays

For FcεRI-mediated mast cell degranulation, BMMCs and PCMCs were sensitized at 37 °C overnight with 2 μg/ml of anti-TNP IgE (BD Biosciences) in RPMI 1640 culture medium containing 30 ng/ml IL-3. Cells were washed three times with Tyrode’s buffer. BMMCs (5 × 10^5^) or PCMCs (3 × 10^5^) in 200 μl Tyrode’s buffer were stimulated at 37 °C with an increasing concentration of TNP-BSA (Biosearch Technologies) as indicated in the figures.

For FcγR-mediated mast cell degranulation, PCMCs were sensitized at 37 °C overnight with 5 μg/ml of rat anti-mouse CD16/32 mAb (clone 2.4G2) (BD Biosciences) in RPMI 1640 culture medium containing 30 ng/ml IL-3. The cell-bound mAb was cross-linked with F(ab’)_2_ fragment of mouse anti-rat IgG (Jackson Immuno Research) in Tyrode’s buffer at 37 °C at the indicated concentrations. After 30 min (for mediator release) or 6 h (for cytokine production), the reactions were terminated by centrifugation at 4 °C. Supernatant were collected and cell pellets were lysed with 1% Triton X-100 in Tyrode’s buffer. Histamine, serotonin, and leukotriene B_4_ (LTB_4_) were measured using the Histamine EIA kit (Bertin Pharma), Serotonin ELISA kit (Enzo Life Sciences), and LTB_4_ ELISA kit (Cayman Chemical), respectively, according to manufacturers’ instructions. Percentage release was calculated as [(supernatant content)/(supernatant content + lysate content)] × 100. TNFα, IL-6, IL-13, CCL-2, and MIP-1α in the supernatant were measured using the Quantikine ELISA kits (R&D Systems) according to the manufacturer’s instructions.

### Neutrophil cell isolation and neutrophil assays

Neutrophils were isolated from bone marrow or peripheral blood using a neutrophil isolation kit (Miltenyi Biotec). For neutrophil bacterial killing assays, two-fold dilutions (2.5–0.625 × 10^6^) of neutrophils were added to 2 × 10^5^ c.f.u. *Escherichia coli* strain 26 and fresh mouse serum (5% final concentration) in a total volume of 1 ml, in triplicate. The tubes were rotated for 2 h and 100 μl of the sample was diluted with 1 ml sterile distilled water (pH adjusted to 11 by NaOH) to lyse cells. Serial dilutions were prepared and plated on LB agar plates without antibiotics. Bacterial colonies were counted after 24 h incubation at 37 °C. Chemotaxis assays were performed by adding 1 × 10^6^ neutrophils in HBSS to the upper chamber of a Transwell unit (3 μm, Costar). The lower chamber was filled with HBSS containing 0.5% bovine serum. After incubating the chambers for 45 min at 37 °C, the transmigrated cells were collected from the lower chamber, fixed, and counted on a flow cytometer. The results are expressed as the fraction of total cells that migrated to the lower chamber. For luminol-based reactive oxygen species measurement, 1 × 10^6^ neutrophils were plated in clear-bottomed 96-well white plates (Corning) in triplicate. The oxidative burst was induced in luminol medium (PBS containing 5 mM glucose, 1 mM MgCl_2_, 0.5 mM CaCl_2_ and 100 μM luminol (Sigma-Aldrich)) by stimulating macrophages with 100 ng ml^−1^ PMA. To blot for neutrophil serine proteases, lysates were prepared by lysing neutrophils with 8 M Urea, 50 mM Tris pH 7.5 buffer, and blotting with antibodies listed in Supplementary Table I.

### OVA-induced allergic peritonitis

Allergic peritonitis was induced as described^56^. In short, 3–4 months old male mice were immunized by subcutaneous injection of 100 μg ovalbumin (OVA) adsorbed in 1.6 mg of alum hydroxide. Seven days later, the mice received the same dose of OVA/alum. Seven days later, peritonitis was induced by intraperitoneal injection of 30 μg of OVA in 300 μl of PBS. The mice were euthanized 1 min after a challenge by CO_2_ inhalation and the peritoneal cavity was lavaged with 5 ml of PBS. Peritoneal lavage fluid was harvested and centrifuged at 4 °C, and supernatants were collected for histamine and serotonin measurements.

### PCA model

Two-to-four months old female mice were shaved on the back one day before sensitization. For IgE-mediated PCA, mice were passively sensitized by intradermal administration of anti-DNP-IgE (clone SPE7, 25 μg in 20 μl of PBS) in the dorsal skin. The mice were challenged 24 h later with an intravenous injection of 200 μg of DNP-OVA containing 1% Evan’s blue solution in 200 μl of PBS via the tail vein. Fifteen minutes later, the mice were euthanized, and Evan’s blue dye was extracted from the dorsal skin at the injection site in formamide. The amount of dye in the extract was determined by measuring the absorbance at 620 nm along with Evan’s blue dye standard curve. For IgG-mediated PCA, the mice were passively sensitized by intradermal injection of anti-TNP-IgG1 (50 μg in 20 μl of PBS) in the dorsal skin. The mice were challenged 5 min later with an intravenous injection of 300 μg of TNP-OVA containing 1% Evan’s blue solution in 200 μl of PBS via the tail vein. The mice were euthanized 2 h later. Evan’s blue dye extraction and measurement were performed as described above.

### K/BxN STA model

K/BxN serum-induced vascular permeability was monitored in vivo by noninvasive near-infrared fluorescence imaging (NIRF) of the mouse whole-paw. Mice were anesthetized by 2% isoflurane (Butler Schein, 1 L/min flow), implanted with a tail vein catheter, and immobilized with the paw secured by surgical tape. For K/BxN experiments depicted in Fig. 6, mice were imaged using the Kodak In-Vivo FX Pro 400 whole-animal NIRF imaging system (Carestream Health). Mice were injected intravenously through the tail vein catheter with 100 μL of the blood pool probe AngioSense 680 (PerkinElmer), imaged at 1-min intervals (650 nm excitation/700 nm emission, 21.4 mm FOV, 10-s exposure, 2× binning) for 5 min to establish baseline fluorescence followed by intravenous injection of 75 μl K/BxN serum and further imaging for another 25 min. For K/BxN experiments depicted in Fig. 6, mice were imaged using the Pearl Imager NIR fluorescence imaging system (Li-COR), with excitation at 700 nm and 85 μm scan resolution. Quantitative image analysis of the mice was performed using Pearl Cam Software (Li-COR). The K/BxN serum used in this study was sourced from KRNxNOD F1 mice that exhibited severe arthritis. The average fluorescence intensities and the fold change in fluorescence intensities from the initial imaging time point within paws were quantified using custom routines in MatLab (MathWorks).

### K/BxN STA and adoptive transfer studies

Wild-type and *Prss57−/−* BMMCs for adoptive transfer were obtained by differentiating bone marrow GMPs in mast cell differentiation media containing IL3 and SCF for 4–5 weeks. The expression of CD117 and FcεRIa was confirmed by flow cytometry. Donor cells were engrafted into 6–8-week old female *Prss57−/−* host mice by intravenous injection (10 × 10^6^ cells in 200 µl of sterile PBS). Wild-type littermate controls were used as a reference group. Donor cells were allowed to engraft for 10 weeks. The induction of vascular leakage in the K/BxN model was performed as described above. For neutrophil engraftment studies, bone marrow-derived neutrophils were aseptically acquired as described above. Donor neutrophils were adoptively transferred into *Prss57−/−* host mice by tail vein injection (2.0 × 10^7^ cells per recipient in 100 µl of sterile PBS). Immediately after the transfer, vascular leakage was induced by K/BxN serum transfer as described above.

### Adoptive transfer into congenic mice

Wild-type and *Prss57−/−* BMMCs were as described above. Upon reaching maturity, a total of 2.5 × 10^6^ donor BMMCs in 200 µl of sterile PBS were intraperitoneally transferred into 6- to 8-week-old-female mice recipients (*Cd45.1*+*/*+ congenic mice; strain B6.SJL-*Ptprc*^*a*^ *Pepc*^*b*^/BoyJ; stock number 002014; The Jackson Laboratory). After 8 weeks of transfer, peritoneal cells were isolated by peritoneal lavage and characterized by flow cytometry. Mast cells were stained with the following antibodies as previously described^54^—PE-Cy5-conjugated anti-lineage antibodies B220 (RA3-6B2), CD3 (17A2), CD4 (GK1.5), CD8b (ebioH35-17.2), CD11b (M1/70), CD19 (eBio1D3), Gr-1 (RB6-8C5), Ter119 (Ter-119), PE-conjugated anti-FceRI (MAR1), FITC-conjugated anti-integrin b7 (FIB504), biotinylated anti-T1/ST2 (DJ8) followed by streptavidin–APC-Cy7, BV 605-conjugated, or Horizon V450-conjugated anti-CD16/32 (2.4G2), Horizon V450 anti-CD127 (SB/199), APC-conjugated anti-CD25 (PC61.5), and Alexa 700-conjugated anti-CD45 (30-F11). To distinguish between recipients (Cd45.1+) and donors (Cd45.2+), cells were stained with Pacific Blue-conjugated anti-Cd45.1 and PE-Cy7-conjugated Cd45.2. Cultured cells were pretreated with anti-CD16/32 (2.4G2) before staining to prevent unspecific binding of antibodies. Antibodies were obtained from BioLegend.

### Histamine-induced vascular leakage

Animals were anesthetized with isoflurane and the body temperature maintained at 37 °C. Imaging was initiated after each animal received a tail vein injection of 2 nM Angiosense 680 fluorescent imaging agent diluted in 150 µl of sterile saline. After 5 min, mice received histamine dihydrochloride (20 mg/kg) intravenously. Animals were imaged over a 20-min period, starting at the time of Angiosense injection and ending 15 min after histamine dihydrochloride administration. After imaging, the tail vein catheter was removed, and the animal was immediately euthanized.

### Ultrastructural analysis of mast cells

Preparations for ultrastructural analysis of mast cells were performed as previously described^57^. Pooled cells from peritoneal lavage of wild-type (*n* = 4 mice) or *Prss57−/−* (*n* = 4 mice) were fixed by immersion fixation in modified Karnovsky’s fixative (2.5% paraformaldehyde and 2% glutaraldehyde in 0.1 M cacodylate buffer, pH 7.2) for at least 24 h at 4 °C. Fixed cells were washed with ultrapure water and post-fixed with 2% aqueous osmium tetroxide for 2 h. The samples were then washed again in ultrapure water and stained “en bloc” with 1% (w/v) uranyl acetate at 4 °C overnight. Following staining, samples were dehydrated in a series of ascending ethanol concentrations, rinsed twice with propylene oxide, and finally embedded in epoxy resin Eponate-12 (Ted Pella, Redding, CA, USA). Semithin sections (500 nm thickness) were cut with the UMC ultramicrotome (Leica Biosystems, Buffalo Grove, IL, USA) using a DIATOME diamond knife for histology (Electron Microscopy Sciences, Hatfield, PA, USA). Sections were transferred to carbon-coated histology glass slides and dried. Finally, sections were stained with 4% aqueous uranyl acetate for 15 min and 0.1% Reynold’s lead citrate for 1 min to enhance contrast^58^. Sections were thoroughly rinsed with ultrapure water and dried on a heat plate before being transferred to the scanning electron microscopy (SEM).

SEM was performed using a GeminiSEM 300 equipped with a field emission gun (Carl Zeiss AG, Oberkochen, Germany). For the operation of the GeminiSEM 300 microscope, the application software SmartSEM (version 6.01) was used (Carl Zeiss AG, Oberkochen, Germany). Imaging was with the backscatter electron detector (BSD1) at 8.5 mm working distance, 30 µm (standard) aperture, 3–6 keV acceleration voltage, and the operation of the field emission gun in “high current” mode. For the majority of images, a scan speed of “5”, noise reduction by 4× line averaging, and an image size of at least 4096 × 3072 (4 k × 3k) pixels were chosen. For imaging of ultrastructural detail pixel sizes between 2 and 5 nm were used. The greyscale of the images was inverted to achieve TEM-like representations. Photoshop CS4 (Adobe) was used to adjust contrast and brightness of whole images, to crop regions of interest, and to reduce the pixel size per area for images that were obtained with a store resolution larger than 4096 × 3072 pixels to produce images at 300 dots per inch (dpi) print resolution for figure preparation. Granule numbers and size (nm) were quantified by ImageJ. Mast cells were distinguished from occasional basophils by the absence of electron-dense cytoplasmic granules representing prominent aggregates of cytoplasmic glycogen^59^.

### Immunofluorescence microscopy for NSP4 cellular localization

Primary cells were fixed with 4% paraformaldehyde for 20 min, washed twice with PBS, quenched with 50 mM ammonium chloride for 10 min, and washed once more with PBS. Fixed cells adhered to polylysine coated chamber slides for 30 min. Cells were then permeabilized for 20 min using permeabilization buffer/blocking buffer containing 0.3% Triton X-100, 5% bovine serum albumin, 2.5% fetal bovine serum, 0.1% tween 20, 0.5% cold fish gelatin and resuspended in PBS. Cells were incubated with primary antibodies (Supplementary Table 2) overnight at 4 °C, followed by repeated washes with blocking buffer. Cells were then incubated with secondary antibodies conjugated with either Alexa Fluor 488 of 647 for 1 h and then mounted with ProLong Gold Antifade Mountant with DAPI (ThermoFisher). Samples were imaged using Axioimager widefield and Leica SP5 confocal microscope.

### RNA sequencing

RNA-sequencing was performed to compare gene expression between primary wild-type and *Prss57−/−* GMPs or BMMCs. RNA was extracted from GMPs or BMMCs using Qiagen RNeasy kit per manufacturer’s recommendations (Qiagen). A quality check of total RNA was done to determine sample quantity and quality. The concentration of RNA samples was determined using NanoDrop 8000 (Thermo Scientific) and the integrity of RNA was determined by Fragment Analyzer (Advanced Analytical Technologies). 0.1 μg of total RNA was used as input material for library preparation using TruSeq Stranded Total RNA Library Prep Kit (Illumina). The size of the libraries was confirmed using 4200 TapeStation and High Sensitivity D1K screen tape (Agilent Technologies) and their concentration was determined by the qPCR-based method using the Library quantification kit (KAPA). The libraries were multiplexed and sequenced on Illumina HiSeq4000 (Illumina) to generate 30 M of single end 50 base pair reads. Processing and analysis of the RNA-sequencing data were performed using the R programming language (http://www.r-project.org) along with packages from the Bioconductor project (http://bioconductor.org). Raw RNA-sequencing reads were processed using the HTSeqGenie Bioconductor package (v. 4.0.1). Briefly, reads were aligned to the reference mouse genome sequence (build 38) using the GSNAP algorithm^60^, and the following parameters: *-M 2 -n 10 -B 2 -i 1 -N 1 -w 200000 -E 1 –pairmax-rna* = *200000 –clip-overlap*. Uniquely aligned reads that fell within exons were counted to give an estimate of expression levels for individual genes. For differential expression analyses, we used the *voom* method to normalize RNA-seq data; differential expression statistics were calculated using the limma package^61–63^.

### MS analysis of endogenous histamine and serotonin

FAC-sorted GMPs from 15 wild-type and 15 *Prss57−/−* mice were pooled into 3 groups per genotype (*n* = 5 mice per group). GMPs were expanded and cultured in mast cell differentiation media for 4 days (to distinguish from primary GMPs, we denote these as mast cell “primed” GMPs). Cells were characterized by flow cytometry to assess the expression of cKIT/FcεRIa. Cells were washed and pellets were flash-frozen and stored at −80 °C prior to analysis. Totally, 800 μL of methanol chilled to −20 °C was added to cell pellets kept on dry ice. Totally, 200 ng of salbutamol was added to each sample as a recovery standard. Samples were homogenized with 0.5 mm zirconium oxide beads in a Bullet Blender tissue homogenizer. Totally, 400 μL of acetonitrile was added to each sample, and the tubes were vortexed and then incubated at −20 °C for 30 min. Samples were centrifuged for ten minutes in a benchtop centrifuge and the supernatants were collected and dried under a vacuum. Each sample was resuspended in 200 μL of acetonitrile.

The LC–MS platform consisted of a Shimadzu Prominence high-performance liquid chromatography (HPLC) coupled to a Thermo LTQ-Orbitrap Velos mass spectrometer. The LC system included two LC20AD pumps, a vacuum degassing system, an autosampler, and a column oven. The HPLC column was a Phenomenex HILIC LC column (2.0 × 100 mm, 3 μm, 100 Å) fitted with a guard cartridge of matching chemistry. Solvent A was 50 mM ammonium formate in water, pH 3.2. Solvent B was acetonitrile. The LC program was: *t* = 0–3 min, 95% B, followed by a linear gradient to 50% B for over 13 min, hold for 5 min at 50% B, then re-equilibration at 95% B for 10 min. The flow rate was 200 μL/min. The LTQ-Orbitrap Velos accurate mass calibration was performed according to the vendor’s instructions. The mass spectrometer was operated in positive ion mode using an electrospray ionization source set to 4.5 kV, sheath gas set to 30 (arbitrary units), and auxiliary gas set to 10 (arbitrary units). The ion transfer tube was maintained at 350 °C, and the S-lens was set to 50%. Full scan MS data was collected between 50 and 700 m/z with the FT analyzer resolution set to 60,000. Higher-energy collisional dissociation MS/MS scans were collected on m/z corresponding to the target analytes with the FT analyzer resolution set to 30,000.

### Heparin analysis

To extract GAGs, cells were homogenized in 500 µL of water containing protease inhibitors [protein estimation on a small portion of the samples was done at this stage], followed by adding an equal volume of 2× pronase digestion buffer (50 mM sodium acetate, pH-6.0). Samples were digested with pronase (Sigma) at a concentration of 0.5 mg/mL at 37 °C for 20 h, with tumbling. The samples were filtered using a 0.45 µm filtering unit and total proteoglycans were purified by diethylaminoethyl cellulose (DEAE)-chromatography. Briefly, pronase digested samples were loaded on a prewashed DEAE-column followed by extensive washing with DEAE-wash buffer (50 mM sodium acetate, 0.2 M NaCl) and eluting the proteoglycans using elution buffer (50 mM sodium acetate, 2.0 M NaCl). Eluted samples were desalted using a PD-10 column equilibrated with 10% aqueous ethanol and the samples were lyophilized. The sample was dissolved in HS-digestion buffer (0.4 M ammonium acetate, 33 mM calcium acetate, pH-7) and digested with Heparin lyase-I, II, and III at 37 °C for 20 h. The digested samples were then passed over a 3 K spin filtering unit (Pall Life Sciences) and the flow-through containing disaccharides was dried using speed-vac. The disaccharides were then tagged with aniline using the reductive coupling method and spiked with a known amount of ^13^C_6_ aniline tagged disaccharide standards prior to running on LCMS. Disaccharide profiling was done using an LTQ-Orbitrap mass spectrometer using a C18 column. The ions were detected in negative mode and quantified by comparing it with the internal standards.

### Western blot

All lysates were prepared with denaturing lysis buffer (8 M Urea, 50 mM Tris pH 8.0). Total protein concentration was determined using the BCA Protein Assay kit (ThermoFisher). Samples were electrophoresed using Novex 4–20% Tris-Glycine gels (ThermoFisher) at 230 V for 40 min and then transferred onto a nitrocellulose membrane using the iBlot Transfer system (ThermoFisher). Membranes were blocked for 1 h with PBS containing 5% non-fat milk and 0.1% Tween 20, followed by two 5-min washes in PBS buffer containing 0.1% Tween 20. Membranes were incubated with primary antibodies (Supplementary Table 1) for 1 h, and secondary horse-radish peroxidase-conjugated antibody for another hour.

### Real-time PCR analysis

RNA isolation was performed using the RNeasy Mini kit (Qiagen). cDNA was generated from RNA using the iScript cDNA Synthesis kit (Bio-Rad). qPCR was performed using the TaqMan Gene Expression Assays (Life Technologies). qPCR reactions were run on a 7900HT Fast Real-Time PCR system (ABI) at the following thermal cycling conditions: holding step of 30 min at 48 °C followed by a holding step of 10 min at 95 °C and 40 cycles of 10 s at 95 °C and 1 min at 60 °C. Gene expression values were calculated by using the 2^−^^ΔCt^ method, normalizing individual transcript levels to housekeeping endogenous control.

### Statistics and reproducibility

All statistical analyses were performed using GraphPad Prism software. Unpaired Student’s *t* test with Welch’s correction was used to compare two sets of data; Unpaired Students *t* test with the Holm–Sidak method was used for multiple comparisons; one-way and two-way ANOVA with Bonferroni’s post hoc test was used to compare multiple groups in one experiment. All scatterplot bars and bar graphs depict means and standard deviation of data. A *p* value of 0.05 or less was considered significant. All data were obtained from at least two biological replicates as indicated in the legends.

### Reporting summary

Further information on research design is available in the Nature Research Reporting Summary linked to this article.

## Supplementary information

Supplementary Information

Description of Additional Supplementary Files

Supplementary Data 1

Reporting Summary

## Data Availability

Authors can confirm that all relevant data, including extended data files and source data files, are included in the paper. All data are available from the corresponding authors on reasonable request. All reagents are available from D.K. or M.V.L.C. under a material transfer agreement with Genentech Inc. The RNAseq datasets has been deposited in NCBI (identifier GSE138697). The source data underlying Figs. 1–7 of the main paper and Supplementary Figs. 1–11 are provided as Supplementary Data 1.

## References

[1] Abraham SN, John ALS (2010). Mast cell-orchestrated immunity to pathogens. Nat. Rev. Immunol..

[2] Arifuzzaman M (2019). MRGPR-mediated activation of local mast cells clears cutaneous bacterial infection and protects against reinfection. Sci. Adv..

[3] Marichal T (2013). A beneficial role for immunoglobulin E in host defense against honeybee venom. Immunity.

[4] Galli SJ, Tsai M (2008). Mast cells: versatile regulators of inflammation, tissue remodeling, host defense and homeostasis. J. Dermatol. Sci..

[5] Pejler G, Wernersson S (2014). Mast cell secretory granules: armed for battle. Nat. Rev. Immunol..

[6] Kunder CA, John ALS, Abraham SN (2011). Mast cell modulation of the vascular and lymphatic endothelium. Blood.

[7] Mukai K, Tsai M, Starkl P, Marichal T, Galli SJ (2016). IgE and mast cells in host defense against parasites and venoms. Semin. Immunopathol..

[8] Binstadt BA (2006). Particularities of the vasculature can promote the organ specificity of autoimmune attack. Nat. Immunol..

[9] Zarbock A, Ley K (2008). Mechanisms and consequences of neutrophil interaction with the endothelium. Am. J. Pathol..

[10] Sadik CD, Kim ND, Iwakura Y, Luster AD (2012). Neutrophils orchestrate their own recruitment in murine arthritis through C5aR and FcγR signaling. Proc. Natl Acad. Sci. USA.

[11] Gill P, Betschel SD (2017). The clinical evaluation of angioedema. Immunol. Allergy Clin..

[12] Cowland JB, Borregaard N (2016). Granulopoiesis and granules of human neutrophils. Immunol. Rev..

[13] Day R, Gorr S-U (2003). Secretory granule biogenesis and chromogranin A: master gene, on/off switch or assembly factor?. Trends Endocrinol. Metab..

[14] Forsberg E (1999). Abnormal mast cells in mice deficient in a heparin-synthesizing enzyme. Nature.

[15] Humphries DE (1999). Heparin is essential for the storage of specific granule proteases in mast cells. Nature.

[16] Duelli A (2009). Mast cell differentiation and activation is closely linked to expression of genes coding for the serglycin proteoglycan core protein and a distinct set of chondroitin sulfate and heparin sulfotransferases. J. Immunol..

[17] Ringvall M (2008). Serotonin and histamine storage in mast cell secretory granules is dependent on serglycin proteoglycan. J. Allergy Clin. Immunol..

[18] Rabenstein DL (2002). Heparin and heparan sulfate: structure and function. Nat. Prod. Rep..

[19] Lawrence R (2008). Evolutionary differences in glycosaminoglycan fine structure detected by quantitative glycan reductive isotope labeling. J. Biol. Chem..

[20] Rabenstein DL, Bratt P, Peng J (1998). Quantitative characterization of the binding of histamine by heparin. Biochemistry.

[21] Chuang W-L, Christ MD, Peng J, Rabenstein DL (2000). An NMR and molecular modeling study of the site-specific binding of histamine by heparin, chemically modified heparin, and heparin-derived oligosaccharides. Biochemistry.

[22] Pham CTN (2006). Neutrophil serine proteases: specific regulators of inflammation. Nat. Rev. Immunol..

[23] Stapels DA, Geisbrecht BV, Rooijakkers SH (2015). Neutrophil serine proteases in antibacterial defense. Curr. Opin. Microbiol..

[24] Adkison AM, Raptis SZ, Kelley DG, Pham CTN (2002). Dipeptidyl peptidase I activates neutrophil-derived serine proteases and regulates the development of acute experimental arthritis. J. Clin. Investig..

[25] Raptis SZ, Shapiro SD, Simmons PM, Cheng AM, Pham CTN (2005). Serine protease cathepsin G regulates adhesion-dependent neutrophil effector functions by modulating integrin clustering. Immunity.

[26] Kessenbrock K (2008). Proteinase 3 and neutrophil elastase enhance inflammation in mice by inactivating antiinflammatory progranulin. J. Clin. Investig..

[27] Perera NC (2012). NSP4, an elastase-related protease in human neutrophils with arginine specificity. Proc. Natl Acad. Sci. USA.

[28] Perera NC (2013). NSP4 is stored in azurophil granules and released by activated neutrophils as active endoprotease with restricted specificity. J. Immunol..

[29] Clark HF (2003). The secreted protein discovery initiative (SPDI), a large-scale effort to identify novel human secreted and transmembrane proteins: a bioinformatics assessment. Genome Res..

[30] Tang T (2010). A mouse knockout library for secreted and transmembrane proteins. Nat. Biotechnol..

[31] Akula S, Thorpe M, Boinapally V, Hellman L (2015). Granule associated serine proteases of hematopoietic cells—an analysis of their appearance and diversification during vertebrate evolution. PLoS ONE.

[32] Lin SJ, Dong KC, Eigenbrot C, van Lookeren Campagne M, Kirchhofer D (2014). Structures of neutrophil serine protease 4 reveal an unusual mechanism of substrate recognition by a trypsin-fold protease. Structure.

[33] Kanani A, Betschel SD, Warrington R (2018). Urticaria and angioedema. Allergy Asthma Clin. Immunol..

[34] Evrard M (2018). Developmental analysis of bone marrow neutrophils reveals populations specialized in expansion, trafficking, and effector functions. Immunity.

[35] Grootens J, Ungerstedt JS, Nilsson G, Dahlin JS (2018). Deciphering the differentiation trajectory from hematopoietic stem cells to mast cells. Blood Adv..

[36] Tsai M (1991). Induction of mast cell proliferation, maturation, and heparin synthesis by the rat c-kit ligand, stem cell factor. Proc. Natl Acad. Sci. USA.

[37] Malbec O (2007). Peritoneal cell-derived mast cells: an in vitro model of mature serosal-type mouse mast cells. J. Immunol..

[38] Nakazawa S (2014). Histamine synthesis is required for granule maturation in murine mast cells. Eur. J. Immunol..

[39] Piliponsky AM (2019). Basophil-derived tumor necrosis factor can enhance survival in a sepsis model in mice. Nat. Immunol..

[40] Xia B, Feasley CL, Sachdev GP, Smith DF, Cummings RD (2009). Glycan reductive isotope labeling for quantitative glycomics. Anal. Biochem..

[41] Ashina K (2015). Histamine induces vascular hyperpermeability by increasing blood flow and endothelial barrier disruption in vivo. PLoS ONE.

[42] Galli SJ, Tsai M (2012). IgE and mast cells in allergic disease. Nat. Med..

[43] Wershil BK, Mekori YA, Murakami T, Galli SJ (1987). 125I-fibrin deposition in IgE-dependent immediate hypersensitivity reactions in mouse skin. Demonstration of the role of mast cells using genetically mast cell-deficient mice locally reconstituted with cultured mast cells. J. Immunol..

[44] Casey FB, Tokuda S (1973). A comparative study of the mechanisms of passive cutaneous anaphylaxis induced by mouse IgG, rabbit IgG, and rabbit F (ab′)2 antibodies. Int. Arch. Allergy Immunol..

[45] Christensen, A. D., Haase, C., Cook, A. D. & Hamilton, J. A. K/BxN serum-transfer arthritis as a model for human inflammatory arthritis. *Front. Immunol.* **7**, 213–229 (2016). 10.3389/fimmu.2016.00213 PMC4889615 27313578

[46] Chen X (2017). Bone marrow myeloid cells regulate myeloid-biased hematopoietic stem cells via a histamine-dependent feedback loop. Cell Stem Cell.

[47] Dy M (1993). Interleukin 3 promotes histamine synthesis in hematopoietic progenitors by increasing histidine decarboxylase mRNA expression. Biochem. Biophys. Res. Commun..

[48] Corbel S, Dy M (1996). Evidence for bidirectional histamine transport by murine hematopoietic progenitor cells. FEBS Lett..

[49] Puri N, Roche PA (2008). Mast cells possess distinct secretory granule subsets whose exocytosis is regulated by different SNARE isoforms. Proc. Natl Acad. Sci. USA.

[50] Kasperkiewicz P, Altman Y, D’Angelo M, Salvesen GS, Drag M (2017). Toolbox of fluorescent probes for parallel imaging reveals uneven location of serine proteases in neutrophils. J. Am. Chem. Soc..

[51] Smithies O, Gregg RG, Boggs SS, Koralewski MA, Kucherlapati RS (1985). Insertion of DNA sequences into the human chromosomal β-globin locus by homologous recombination. Nature.

[52] Gertsenstein M (2010). Efficient generation of germ line transmitting chimeras from C57BL/6N ES cells by aggregation with outbred host embryos. PLoS ONE.

[53] Monoclonal Antibodies, N. R. C. (US) C. on M. of P. *Generation of Hybridomas: Permanent Cell Lines Secreting Monoclonal Antibodies*. (National Academies Press, US, 1999).

[54] Dahlin JS, Ding Z, Hallgren J (2015). Distinguishing mast cell progenitors from mature mast cells in mice. Stem Cells Dev..

[55] Jensen Bettina M, Swindle Emily J, Shoko Iwaki, Gilfillan Alasdair M (2006). Generation, isolation, and maintenance of rodent mast cells and mast cell lines. Curr. Protoc. Immunol..

[56] Zuany-Amorim C, Leduc D, Vargaftig BB, Pretolani M (1993). Characterization and pharmacological modulation of antigen-induced peritonitis in actively sensitized mice. Br. J. Pharm..

[57] Reichelt M, Sagolla M, Katakam AK, Webster JD (2020). Unobstructed multiscale imaging of tissue sections for ultrastructural pathology analysis by backscattered electron scanning microscopy. J. Histochem. Cytochem..

[58] Reynolds ES (1963). The use of lead citrate at high ph as an electron-opaque stain in electron microscopy. J. Cell Biol..

[59] Galli SJ, Dvorak AM, Dvorak HF (1984). Basophils and mast cells: morphologic insights into their biology, secretory patterns, and function (Part 1 of 7). Mast. Cell Act. Mediat. Release.

[60] Wu TD, Nacu S (2010). Fast and SNP-tolerant detection of complex variants and splicing in short reads. Bioinformatics.

[61] Ritchie ME (2015). limma powers differential expression analyses for RNA-sequencing and microarray studies. Nucleic Acids Res..

[62] Phipson B, Lee S, Majewski IJ, Alexander WS, Smyth GK (2016). Robust hyperparameter estimation protects against hypervariable genes and improves power to detect differential expression. Ann. Appl. Stat..

[63] Law CW, Chen Y, Shi W, Smyth G (2014). K. voom: precision weights unlock linear model analysis tools for RNA-seq read counts. Genome Biol..

