# Supplementary material for: Neutrophil serine protease 4 is required for mast cell-dependent vascular leakage
Source: Commun Biol. 2020 Nov 19;3:687. doi: 10.1038/s42003-020-01407-0 (PMC7677402; doi:10.1038/s42003-020-01407-0)

Supplementary Information for

**Neutrophil Serine Protease 4 is required for mast cell-dependent vascular leakage**

**Authors:** Andrew P. Ah Young^1^†, Sterling C. Eckard^2^†, Alvin Gogineni^3^, Hongkang Xi^2^, S. Jack Lin^1^, Stefan Gerhardy^1^, Christian Cox^2^, Qui T. Phung^4^, Jason A. Hackney^5^, Anand Kumar Katakam^6^, Mike Reichelt^6^, Patrick Caplazi^6^, Paolo Manzanillo^2^, Juan Zhang^7^, Merone Roose-Girma^8^, Lucinda W. Tam^8^, Robert J. Newman^8^, Aditya Murthy^9^, Robby M. Weimer^3^, Jennie R. Lill^4^, Wyne P. Lee^7^, Michele Grimbaldeston^10^, Daniel Kirchhofer^1*^, Menno van Lookeren Campagne^2, 11*^

**This file includes:**

Supplementary Figures 1-12

Supplementary Tables 1-2

Supplementary Figure 12 contains all uncropped western blots for main and supplementary figures.

**
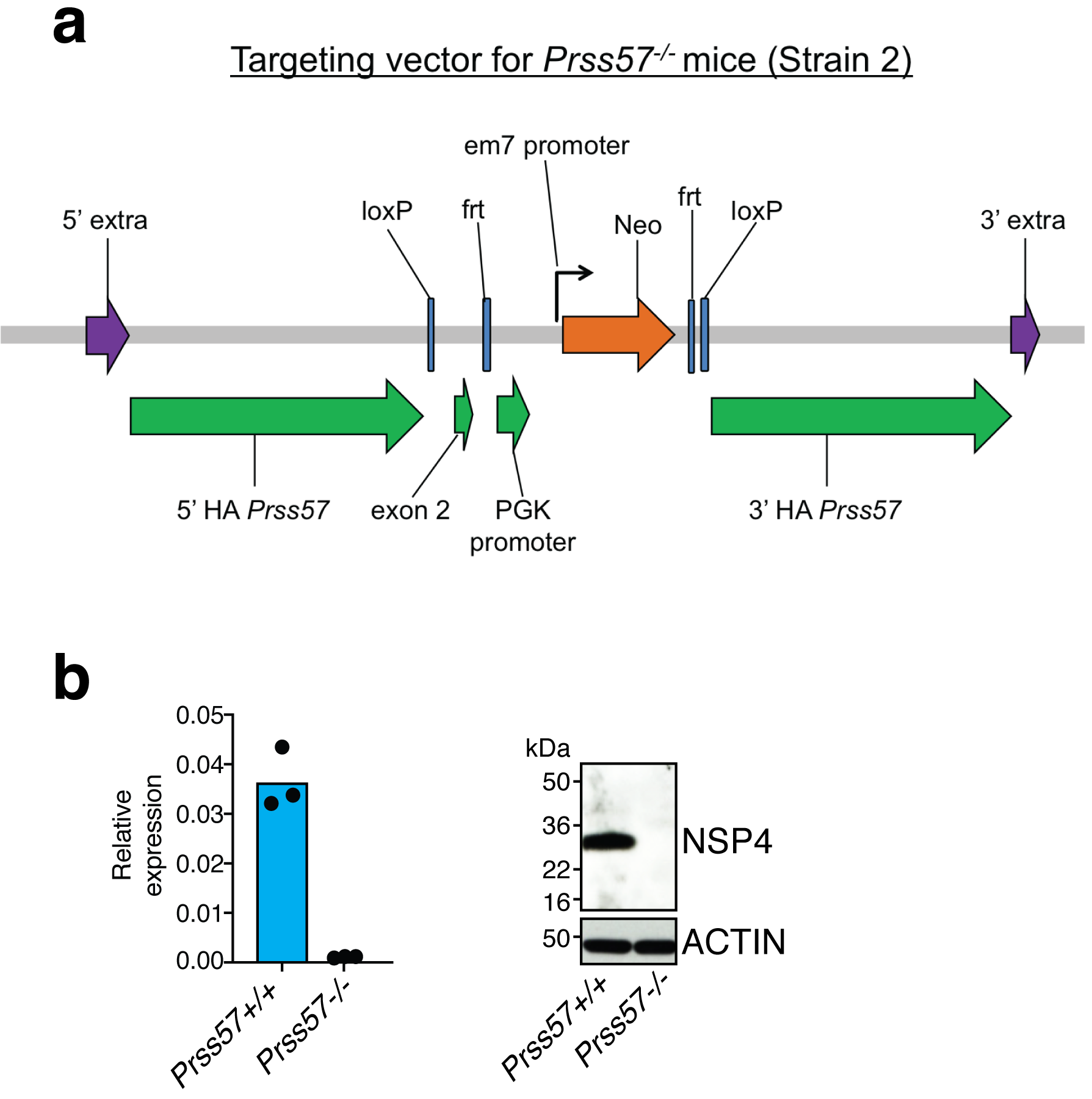
**

**Supplementary Figure 1. Generation and characterization of NSP4-deficient (*Prss57-/-*) mice**

(a) Targeting vector for generating germline *Prss57-/-* mice (Strain 2). Strain 2 mice were used for all

experiments, except for experiments in Fig. 6, which used *Prss57-/-* mice (Strain 1). (b) Quantitative PCR and western blot for NSP4 expression in the bone marrow; mRNA expression is normalized to housekeeping gene Hprt1. Data are presented as mean; n = 3 biological replicates per genotype. Data shown are representative of three independent experiments.

**
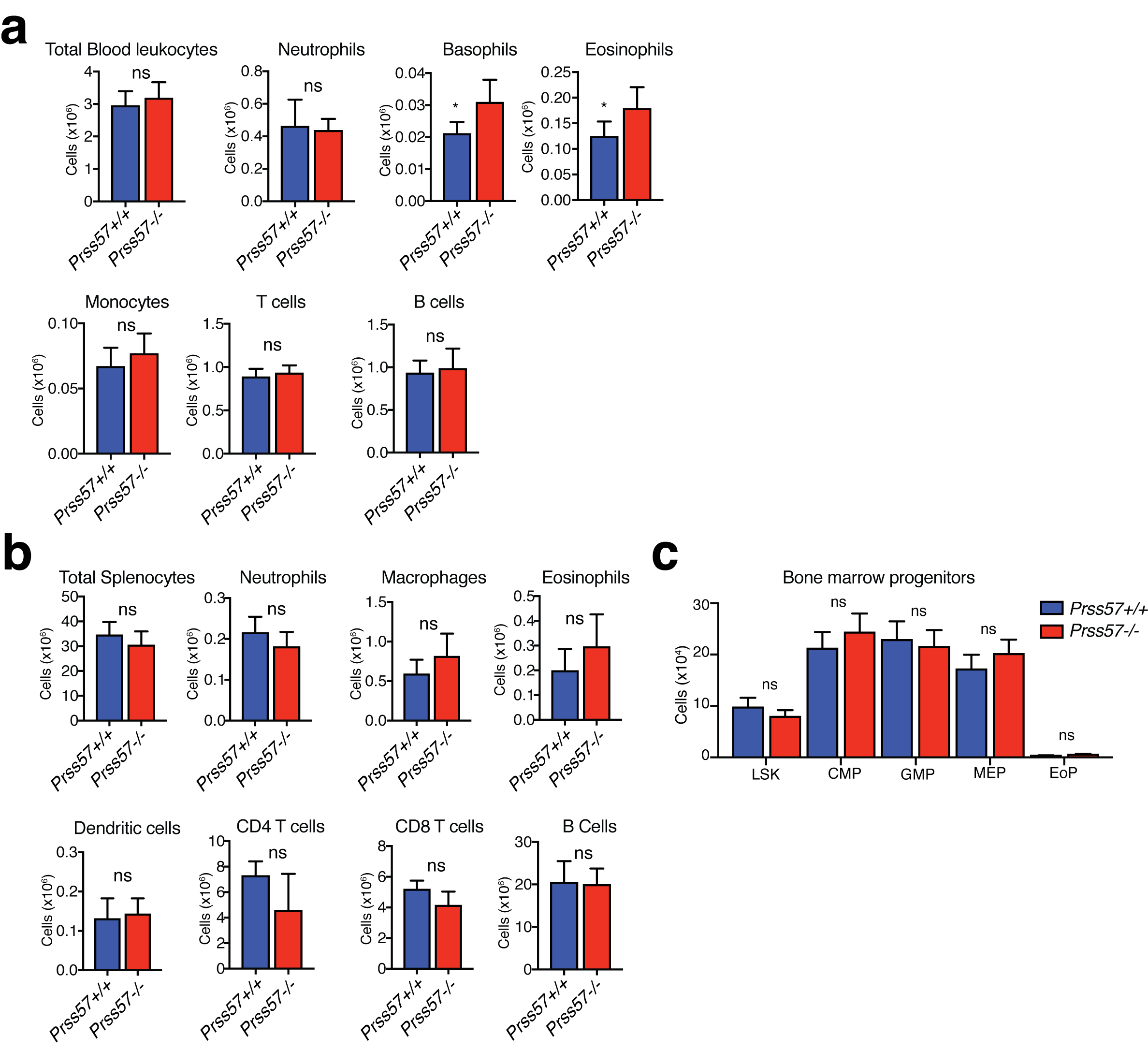
**

**Supplementary Figure 2. Effect of NSP4 deletion on leukocyte populations in the peripheral blood, spleen, and bone marrow**

Flow cytometry characterization of leukocyte sub-populations isolated from the peripheral blood (a), spleen (b) and bone marrow (c) of *Prss57+/+* and *Prss57-/-* littermates. LSK: Lin-, SCA-1-, CD117+ cells; Common myeloid progenitors (CMP): Lin- SCA-1-, CD117+, CD34+, CD16/32^lo^; Granulocyte-macrophage progenitors (GMP): Lin-, SCA-1-, CD117+, CD34+, CD16/32^hi^; Megakaryocyte/erythrocyte progenitors (MEP): Lin-, SCA-1-, CD117+, CD34-, CD16/32^lo^; eosinophil progenitors (EoP). Data are presented as mean ± s.d.; n = 5-6 biological replicates per genotype; **P*<0.05; Student’s t-test. Data shown are representative of three independent experiments.

**
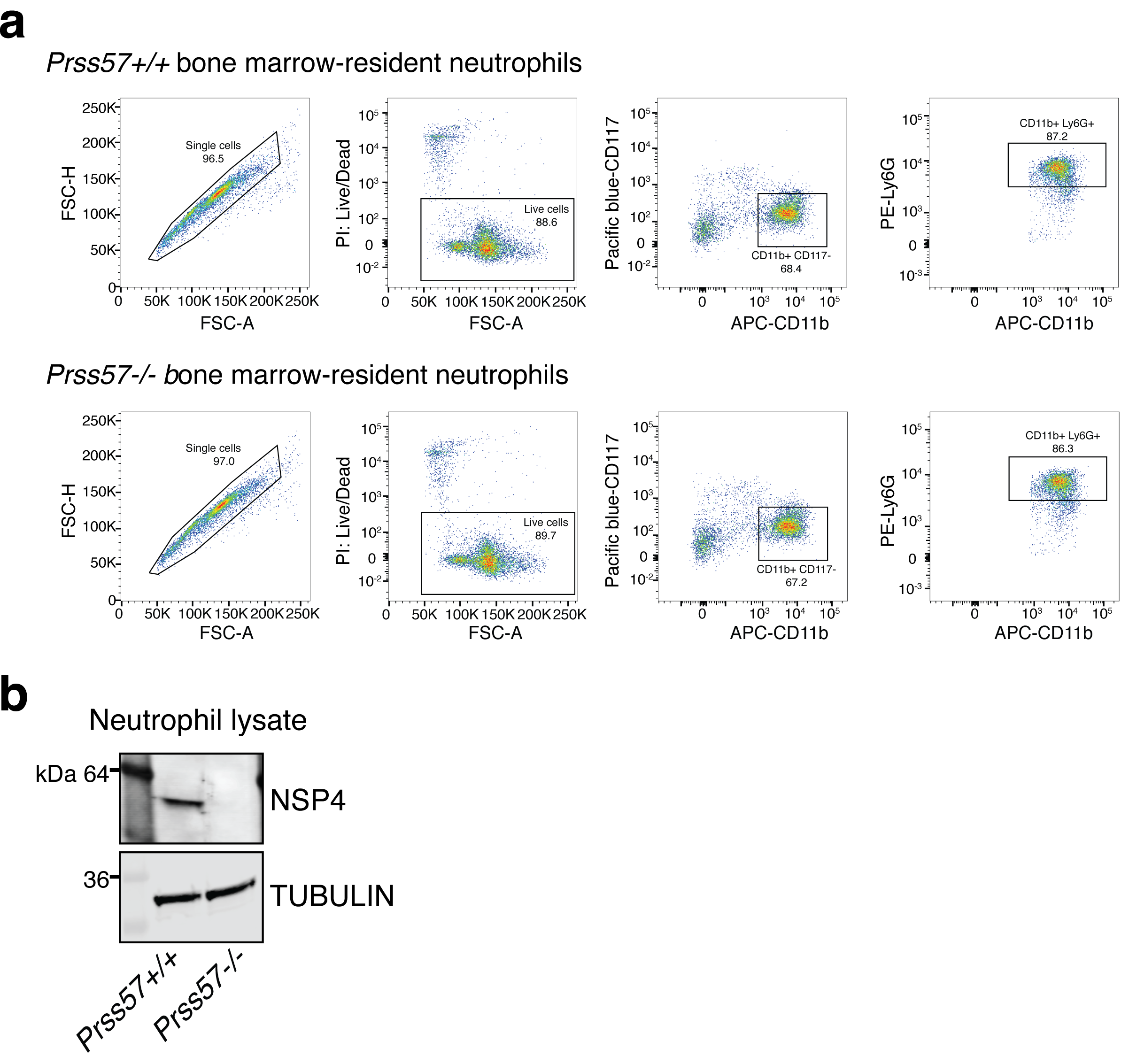
**

**Supplementary Figure 3. NSP4 is expressed in bone marrow-resident neutrophils**

(a) Representative FAC-sorting strategy for the isolation of bone marrow-resident neutrophils from *Prss57+/+* and *Prss57-/-* littermates. (b) Western blot of NSP4 in lysates prepared from FAC-sorted, bone marrow-resident neutrophils. TUBULIN was used as a loading control. Data shown are representative of three independent experiments

**
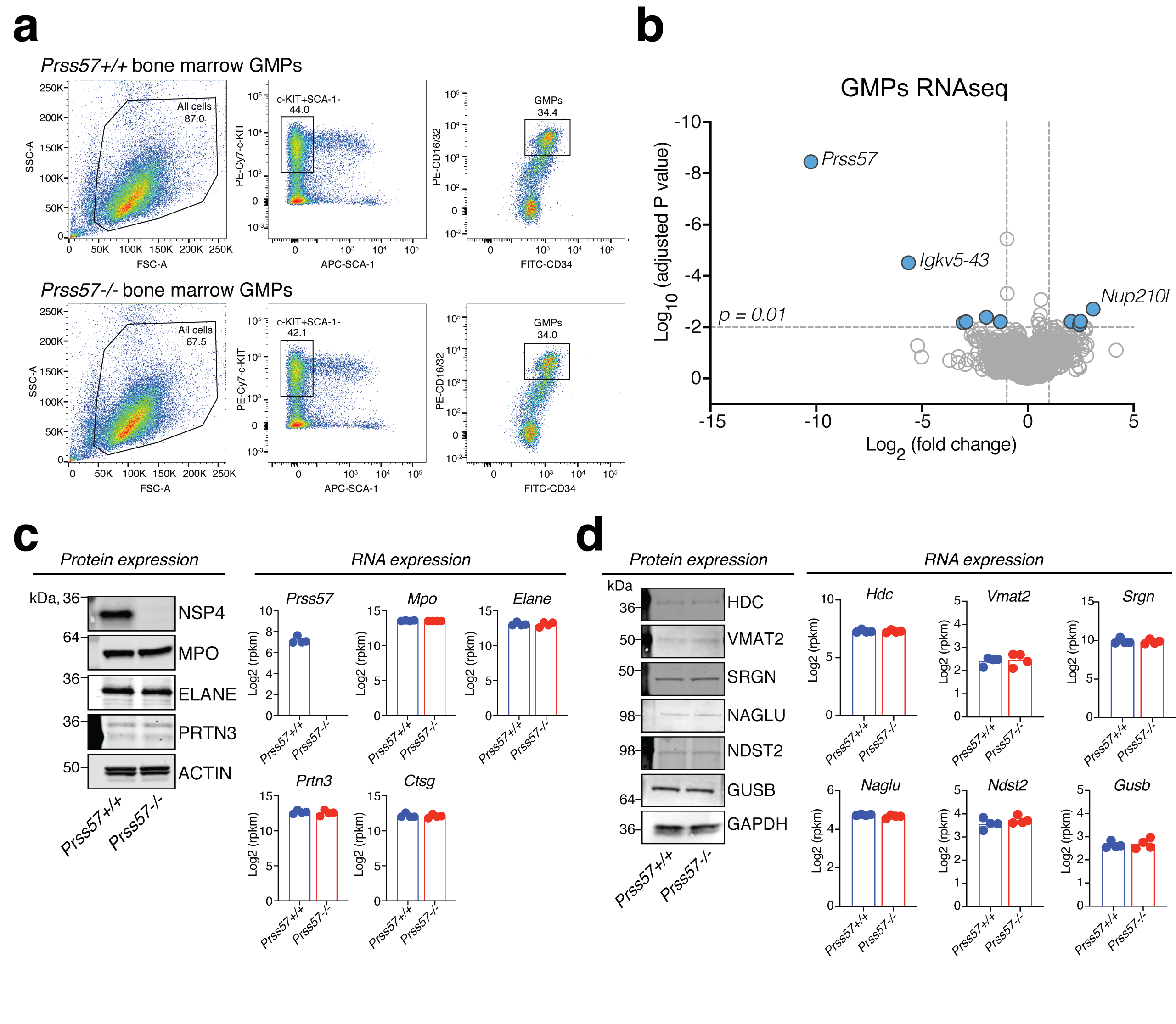
**

**Supplementary Figure 4. Transcriptome profiling and protein expression validation in primary GMPs**

(a) Representative FAC-sorting strategy for the isolation of primary GMPs from the bone marrow (Lin-, SCA-1-, CD117+, CD34+, CD16/32^hi^) of wildtype and *Prss57-/-* littermates. (b) Volcano plot indicating the fold-change (represented as log2 (fold-change)) in mRNA expression in *Prss57+/+* versus *Prss57-/-* GMPs. Differentially expressed genes (blue circles) are based on the following cutoff: adjusted p-value <0.01 and fold-change of 2 or greater. A complete list of differentially expressed genes is shown in Table 1. (c) Western blot and mRNA expression of neutrophil serine proteases and myeloperoxidase in lysates prepared from FAC-sorted primary GMPs (e.g. myeloperoxidase (MPO), elastase (ELANE), and proteinase 3 (PRTN3)). ACTIN was used as a loading control. (d) Western blot and mRNA expression of proteins involved in pathways associated with histamine biosynthesis (e.g. HDC), transport and storage (e.g. VMAT2 and SRGN) and heparin biosynthesis (e.g. NDST2). Gapdh was used as a loading control. mRNA expression data in figures (c) and (d) are obtained from RNA sequencing analysis of primary GMPs shown in (b) (Accession number: GSE138697). Data are presented as mean, n = 4 biological replicates per genotype; Student’s t-test. Western blots in figures (c) and (d) are representative of three independent experiments.

**
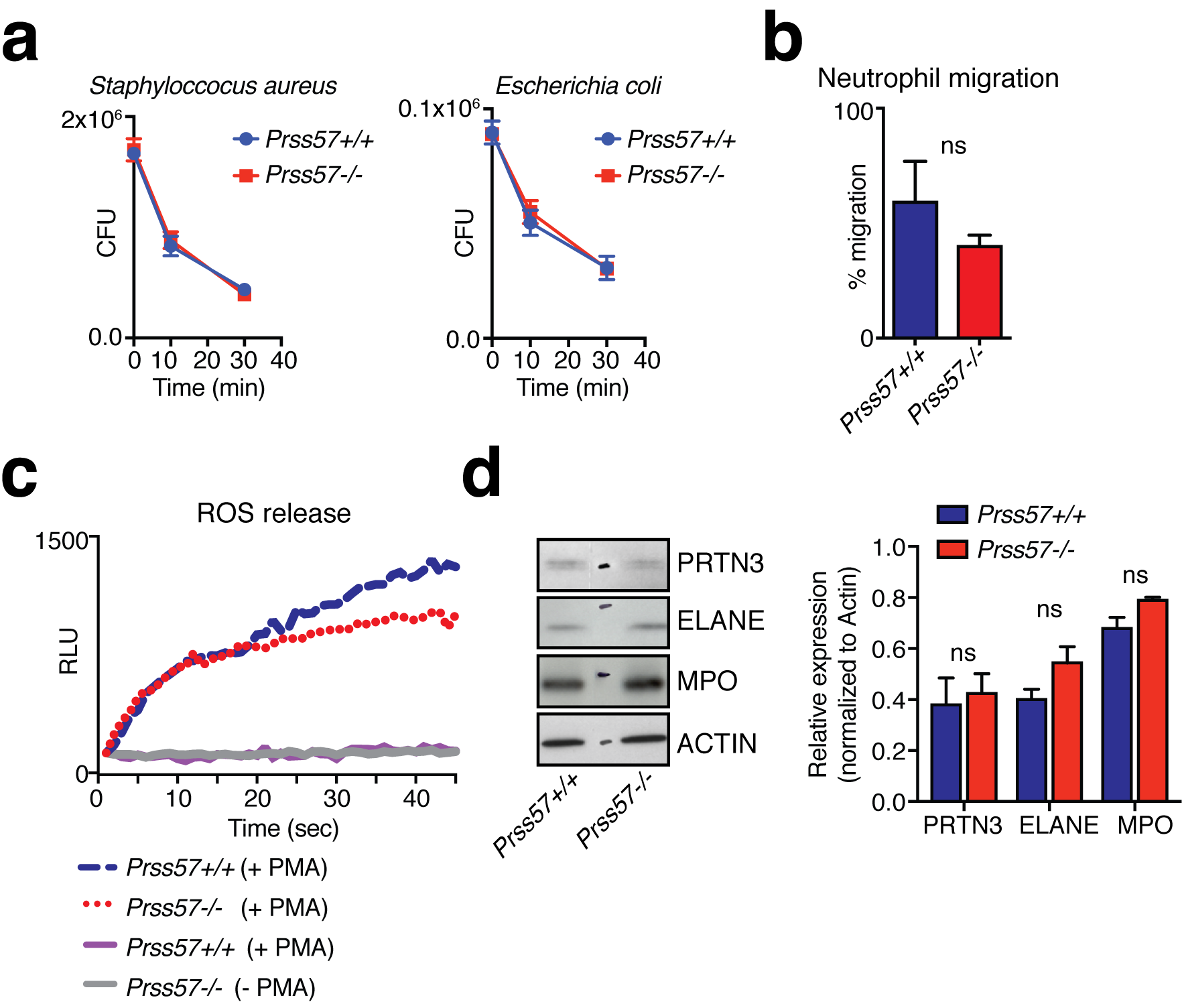
**

**Supplementary Figure 5. NSP4 is not required for neutrophil function**

(a) Bacterial killing by *Prss57+/+* and *Prss57-/-* bone marrow-resident neutrophils isolated from wildtype and *Prss57-/-* littermates; colony forming units (CFU) presented as mean ± s.d. (n = 3 biological replicates per genotype). (b) Neutrophil migration in response to bovine serum. Data are presented as mean ± s.d.; n = 2 biological replicates per genotype; Student’s t-test. (c) Time-dependent release of reactive oxygen species (ROS) upon stimulation with phorbol-12-myristate-13-acetate (PMA). ROS levels were measured by the chemiluminescent oxidation of luminol and expressed as relative luminescence units (RLU). Data are presented as mean ± s.d.; n = 2-3 biological replicates per genotype; two-way ANOVA and Bonferroni post-hoc test. (d) Western blot for neutrophil serine proteases in bone marrow-derived neutrophils (e.g. Proteinase 3 (PRTN3), neutrophil elastase (ELANE) and myeloperoxidase (MPO). ACTIN was used as a loading control. Relative quantification of protein levels based on the semi-quantitative analysis of band intensity and normalization to an ACTIN loading control. Data are presented as mean ± s.d.; n = 3 biological replicates per genotype; Student’s t-test. Data shown are representative of at least two independent experimental repeats.

**
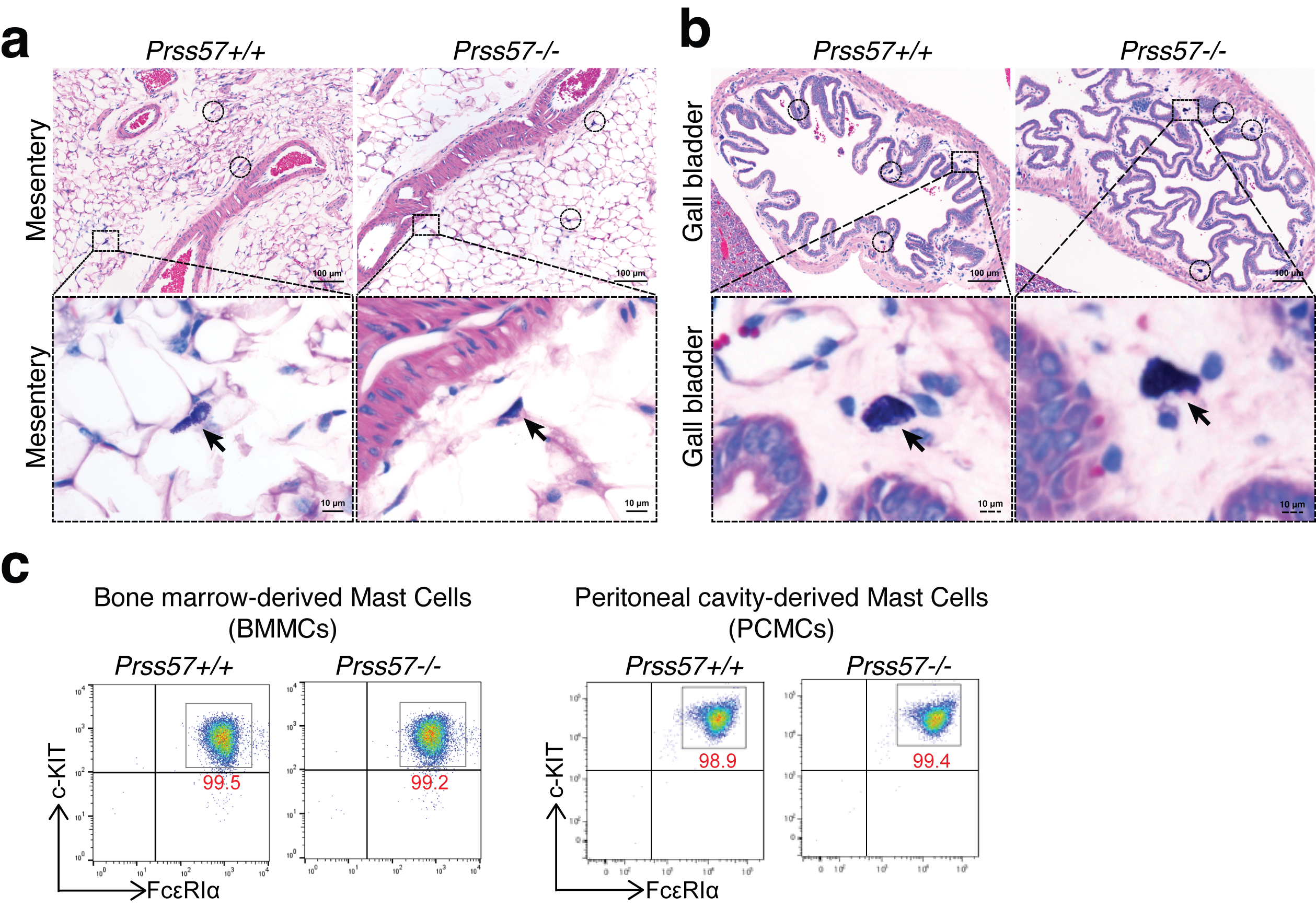
**

**Supplementary Figure 6. Effect of NSP4 on mast cell tissue distribution and *in vitro* differentiation**

Giemsa-stained sections of the mesentery (a) and gall bladder (b) showing general mast cell distribution and homing in *Prss57+/+* and *Prss57-/-* littermates. Mast cells are circled. Mast cells present in the mesentery represent connective-tissue mast cells (CTMCs), and those in the gall bladder represent mucosal mast cells (MMCs). At high magnification, mast cells (arrows) in both genotypes are characterized as elongate, angular cells, approximately 15-20 μm long and 8 μm wide. Their cytoplasm is densely packed with dark purple granules that obscure the nuclei. (c) Representative flow cytometry plots of *in vitro* differentiated BMMCs and PCMCs after 4 weeks of differentiation and stained for surface expression of the mast cell receptors, CD117 (also known as c-KIT) and FcεRIα. Data shown are representative of at least two independent experimental repeats.

**
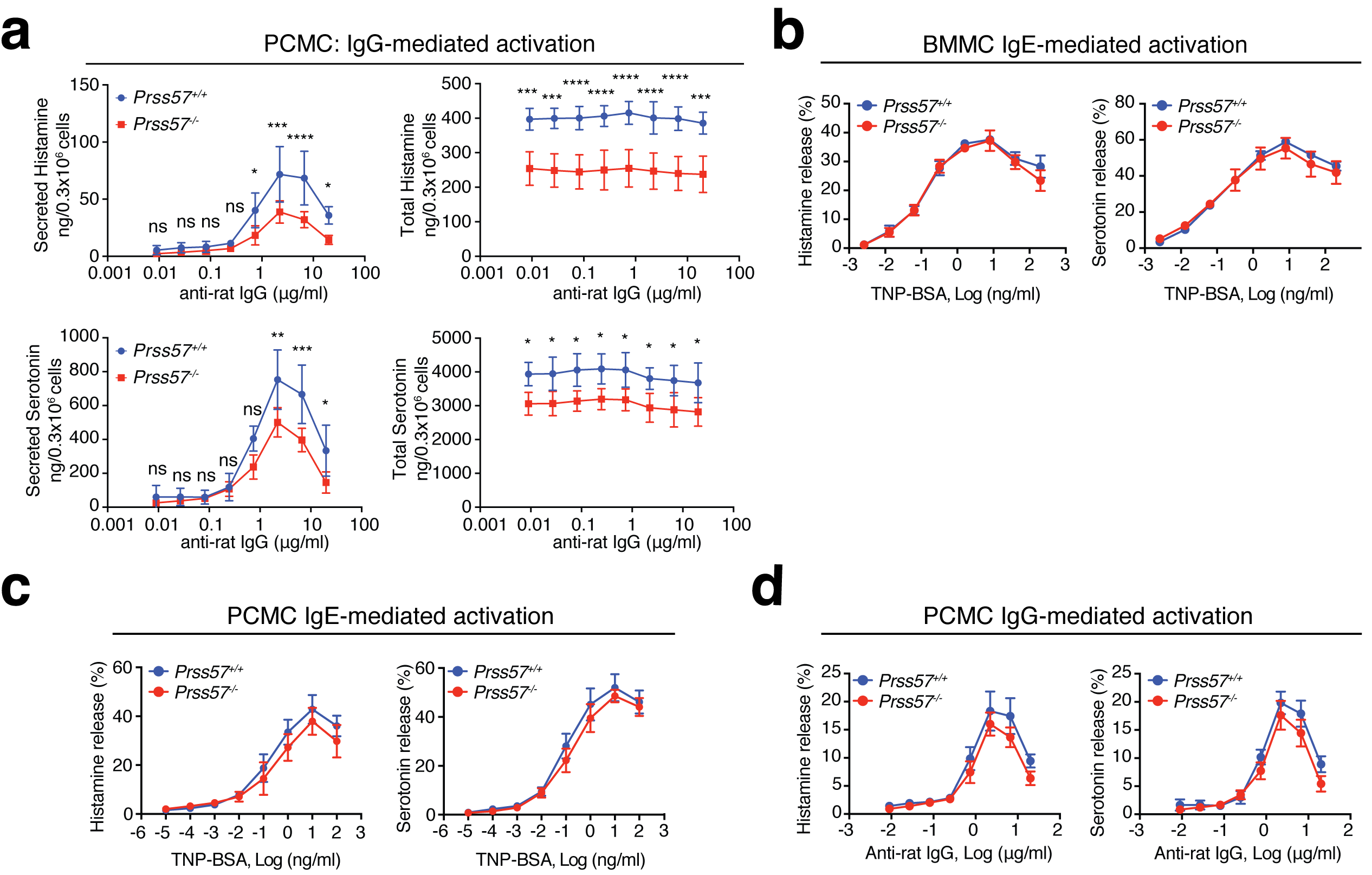
**

**Supplementary Figure 7. Effect of NSP4 on histamine and serotonin levels and release by PCMCs and BMMCs**

Comparing histamine and serotonin levels in *Prss57+/+* and *Prss57-/-* mast cells isolated from wildtype and *Prss57-/-* littermates. (a) Total or secreted levels of histamine and serotonin following IgG-mediated activation of PCMCs. Data are represented as mean ± s.d., n = 4 biological replicates per genotype; **P*<0.05, ***P*<0.01, ****P<*0.001, *****P<*0.0001; two-way ANOVA and Bonferroni post-hoc test. (b) Percentage of histamine or serotonin release by IgE-mediated degranulation of BMMCs and (c) PCMCs, and IgG-mediated degranulation of (d) PCMCs. The percentage (%) of release was calculated by dividing the amount of secreted histamine or serotonin (supernatant levels) to the total amount (supernatant + lysate levels) and multiplied by 100. Data are presented as mean ± s.d.; n = 5 biological replicates per genotype; two-way ANOVA and Bonferroni post-hoc test. Data shown are representative of at least three independent experimental repeats.

**
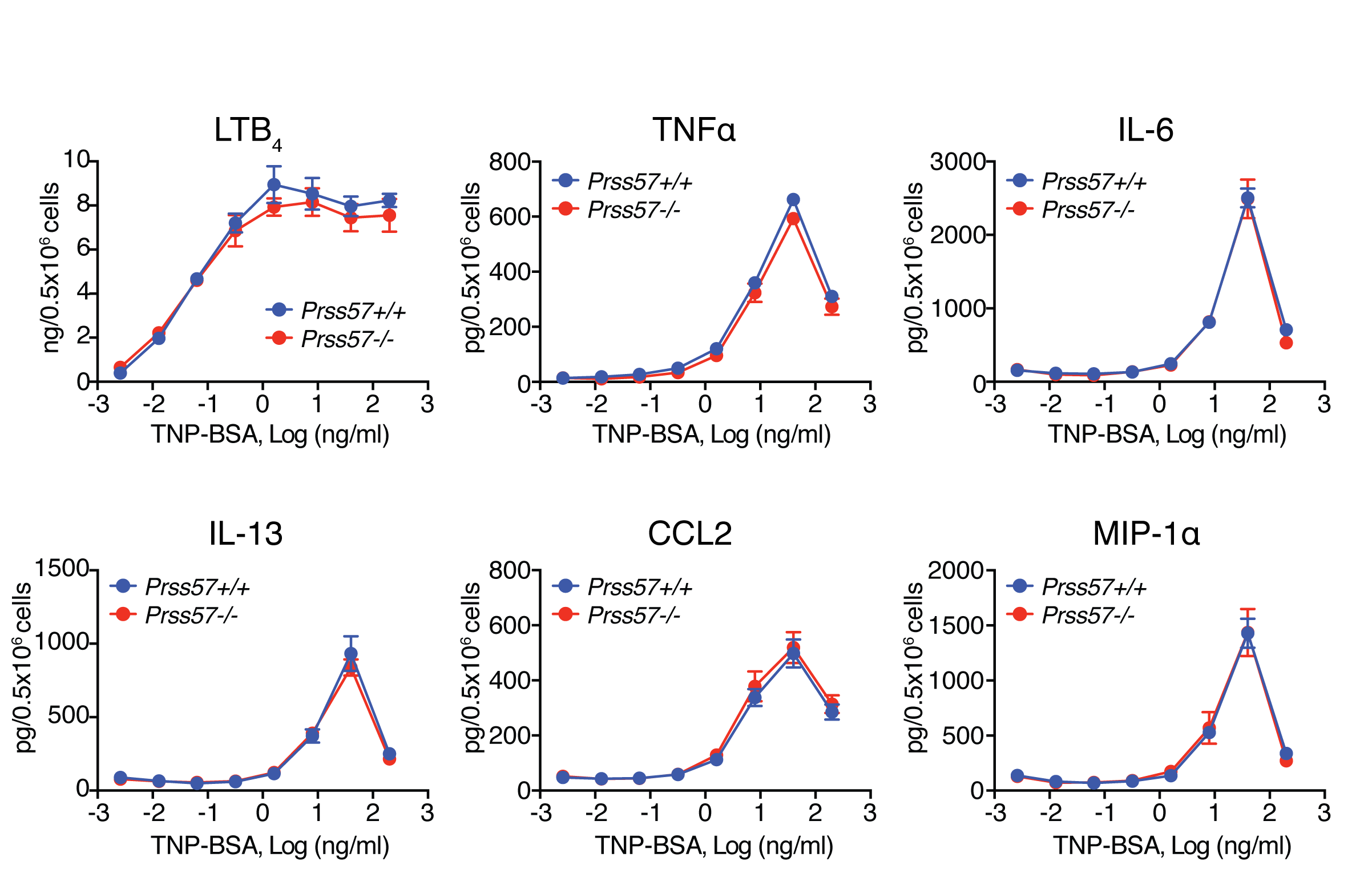
**

**Supplementary Figure 8. NSP4 is not required for the IgE-mediated release of leukotriene B4 (LTB4) and various cytokines from BMMCs**

Quantification of LTB4 and various cytokines and chemokines following IgE-mediated activation of *Prss57*+/+ and *Prss57*-/- BMMCs (isolated from littermates). Data are presented as mean ± s.d.; n = 5 biological replicates per genotype; two-way ANOVA and Bonferroni post-hoc test. Data shown are representative of at least three independent experimental repeats.

**
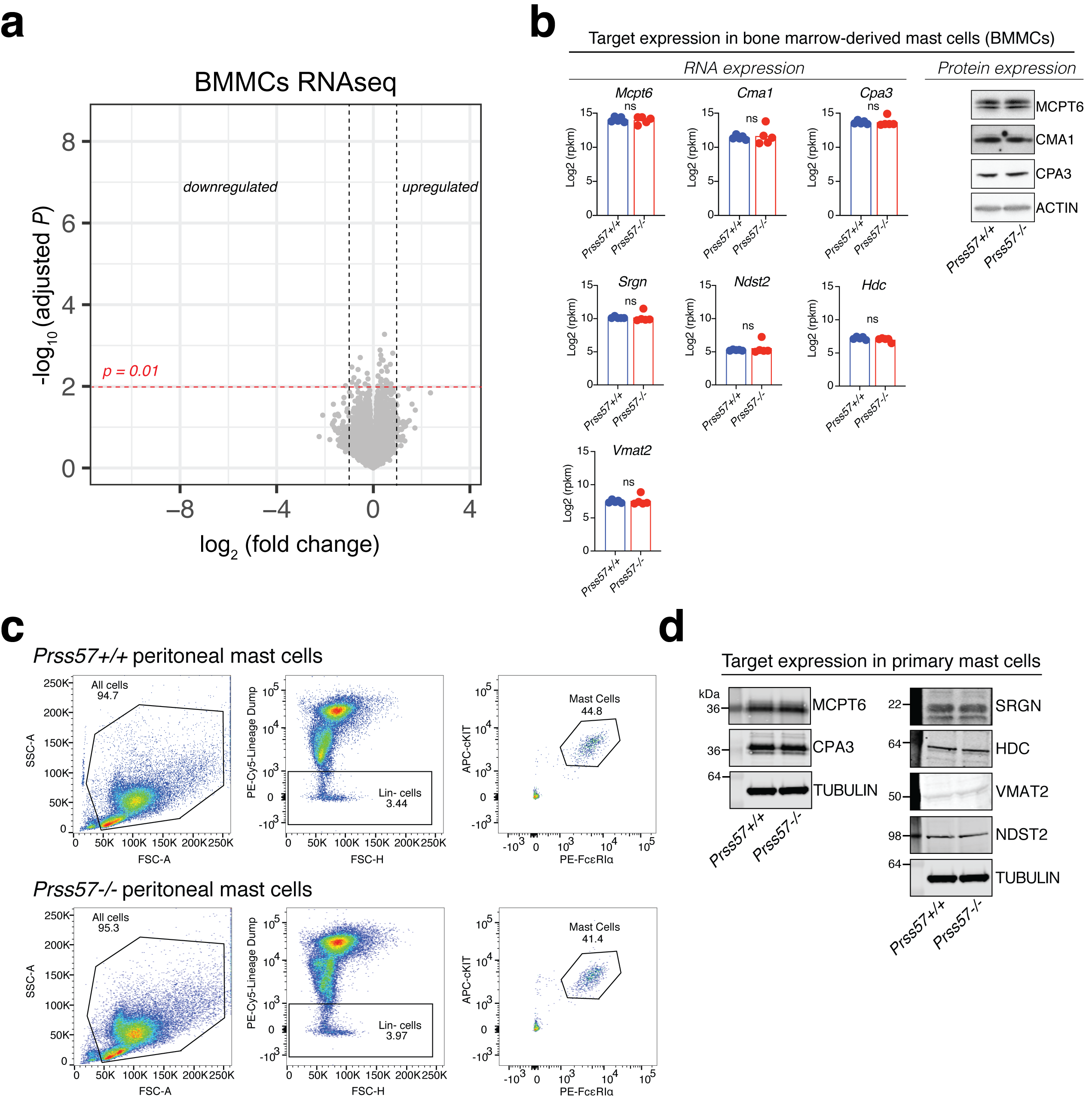
**

**Supplementary Figure 9. Effect of NSP4 on gene expression in primary and *in vitro*-derived mast cells**

(a) Volcano plot indicating the fold-change in mRNA expression in *Prss57+/+* versus *Prss57-/-* BMMCs (littermates). Data represented as log_2_ (fold-change). (b) mRNA expression of the indicated genes based on RNA sequencing data in fig. (a) (Accession number: GSE138697). Data are presented as mean; n = 5 biological replicates per genotype; Student’s t-test. Representative western blots showing protein expression of mast cell proteases (MCPT6, CMA1, and CPA3) in lysates prepared from BMMCs. (c) Representative FAC-sorting strategy for the isolation of peritoneal mast cells (defined by CD117 (c-KIT) and FcεRIα expression). (d) Western blot of mast cell proteases (e.g. tryptase (MCPT6) and carboxypeptidase A3 (CPA3)) and proteins associated with histamine biosynthesis, transport and storage (HDC, VMAT2, SRGN) and heparin biosynthesis (NDST2) in lysates prepared from FAC-sorted primary peritoneal mast cells. Western blots in figures (c) and (d) are representative of at least two independent experimental repeats.

**
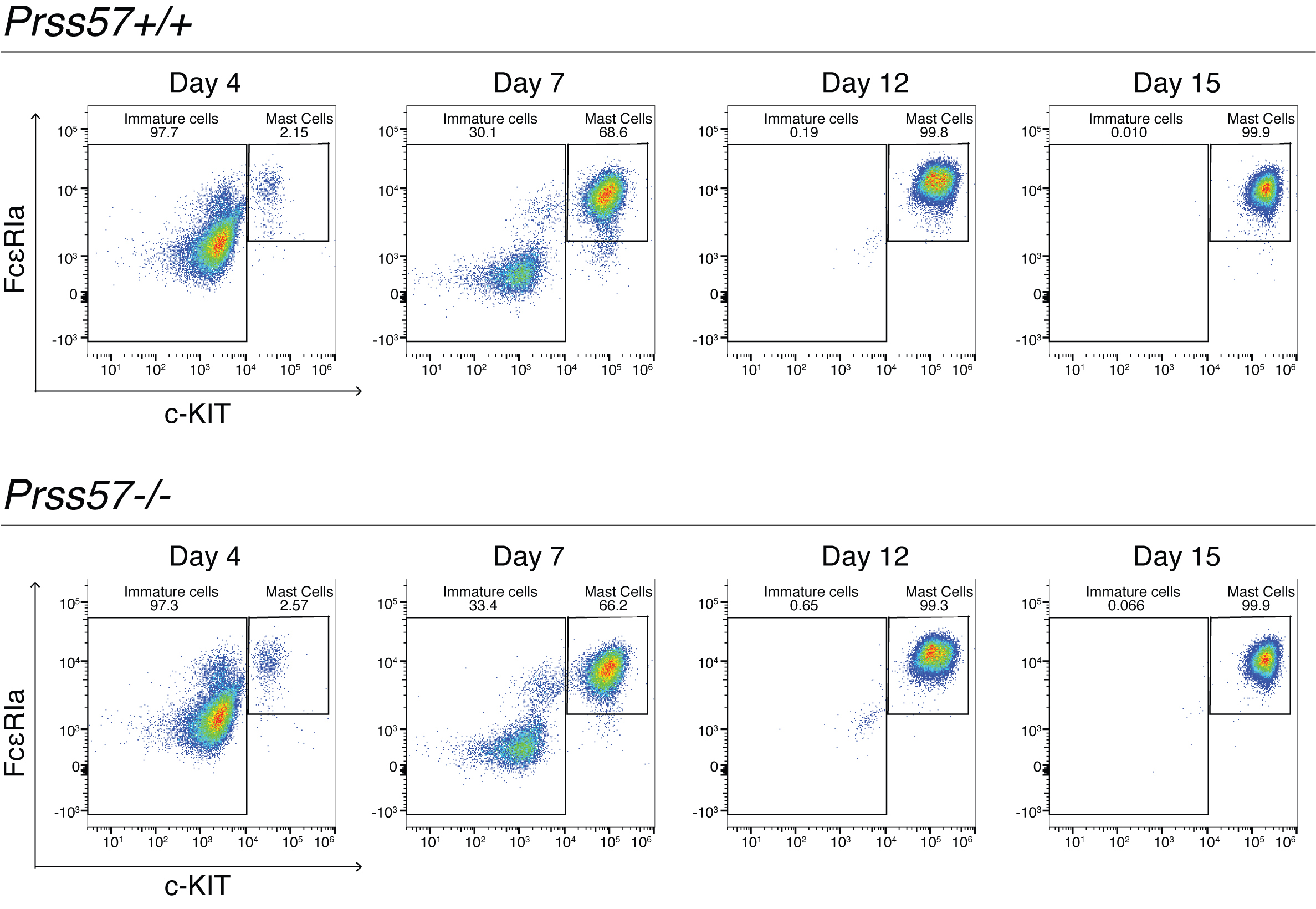
**

**Supplementary Figure 10. Time-course of GMP differentiation into mast cells**

Flow cytometry analysis of FAC-sorted GMPs (Fig. S4a shows FAC-sorting strategy for the isolation of primary GMPs) undergoing mast cell differentiation at the indicated time-points (from day 4-15) in culture media containing IL3 and SCF. Mast cells are defined by the expression of CD117 (c-KIT) and FcεRIα. Data shown are representative of at least two independent experimental repeats.

**
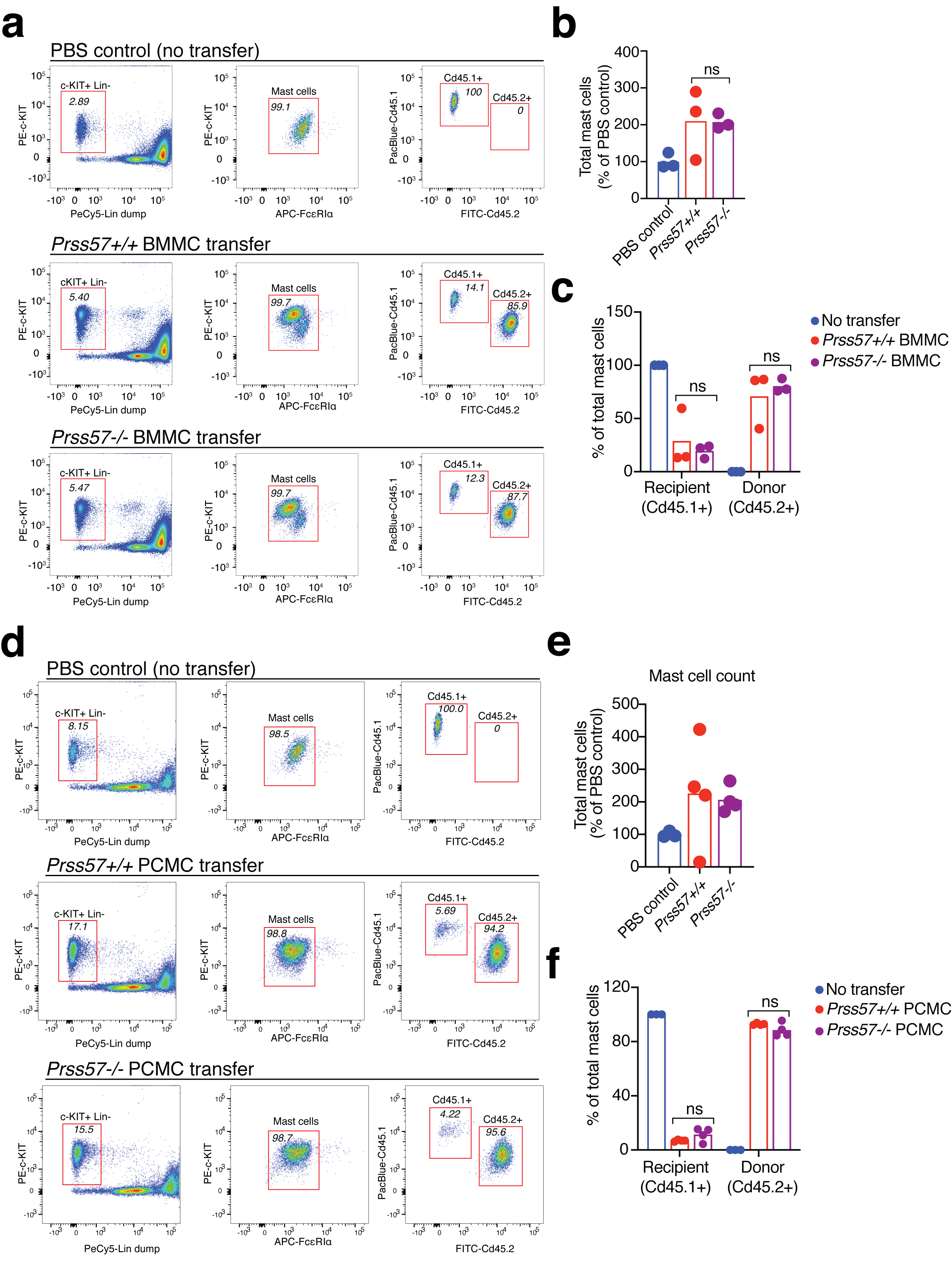
**

**Supplementary Figure 11. Effect of NSP4 on the adoptive transfer efficiency of BMMCs and PCMCs**

(a) Representative flow cytometric analysis of mast cells isolated by peritoneal lavage of *Cd45.1+/+* recipients that received PBS alone (No transfer control; top panel), or donor *Prss57+/+* BMMCs (*Cd45.2+/+;* middle panel), or *Prss57-/-* BMMCs (*Cd45.2+/+;* bottom panel). BMMCs were derived from *Prss57+/+* and *Prss57-/-* littermates. (b) Quantification of total mast cells in the peritoneum (total mast cells = *Cd45.1+/+* recipient plus *Cd45.2+/+* donor mast cells). Data are presented as mean; n = 3 biological replicates per genotype; Student’s *t*-test. (c) Quantification of recipient or donor mast cells present in the peritoneum of recipient mice. Data are presented as mean; n = 3 biological replicates per genotype; two-way ANOVA and Bonferroni post-hoc test. Data shown are representative of at least two independent experimental repeats. (d) Representative flow cytometric analysis of mast cells isolated by peritoneal lavage of *Cd45.1+/+* recipients that received PBS alone (no transfer control; top panel), or donor *Prss57+/+* PCMCs (*Cd45.2+/+;* middle panel), or *Prss57-/-* PCMCs (*Cd45.2+/+;* bottom panel). (e) Quantification of mast cells in the peritoneum. Data are presented as mean; n = 3-4 biological replicates per genotype; Student’s *t*-test. (f) Quantification of recipient or donor mast cells present in the peritoneum of recipient mice. Data are presented as mean; n = 3-4 biological replicates per genotype; two-way ANOVA and Bonferroni post-hoc test. Data shown are representative of at least two independent experimental repeats.

**Supplementary Table 1. Differentially expressed genes based on RNA sequencing of *Prss57+/+* and *Prss57-/-* GMPs**

List of differentially expressed genes in *Prss57*-/- GMPs based on an adjusted p-value cutoff of 0.01 or less, and a relative fold-change of 2 or greater in mRNA expression.

**Supplementary Table 2. Antibodies used in present study**

List of antibodies used for immunohistochemistry and western blot assays. Refer to Methods section for a complete list of flow cytometry antibodies.

**Supplementary Figure 12. Uncropped western blots for main and supplementary figures as indicated.**

Main figures: 1b, 4a

Supplementary figures: 1b, 3b, 4c, 4d, 5b, 9b, 9d

Figure 1b. Western blot for NSP4 expression in primary GMP and CMP lysates


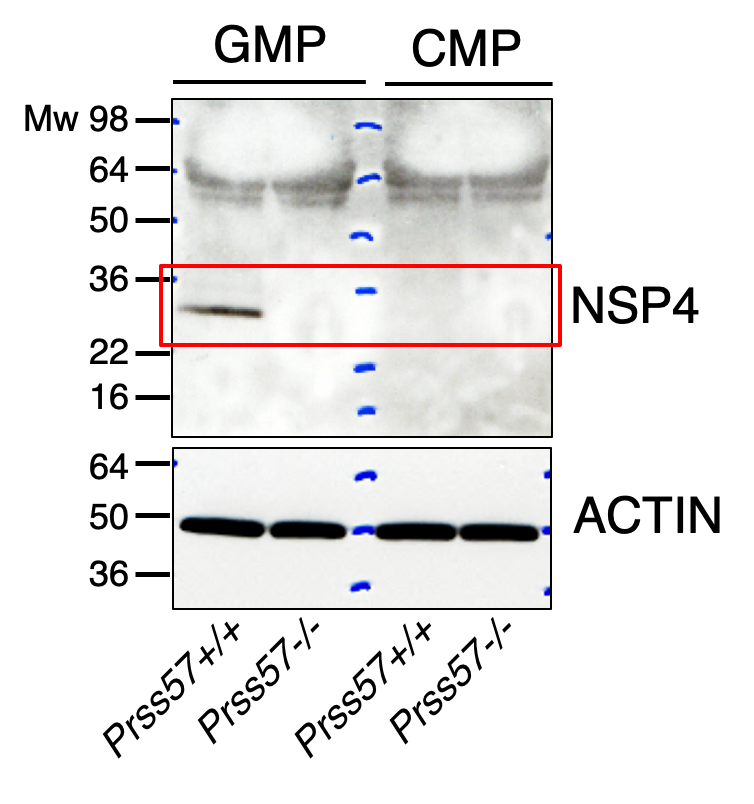


Figure 4a. Western blot for NSP4 and Tryptase B2 expression during GMP-to-mast cell differentiation


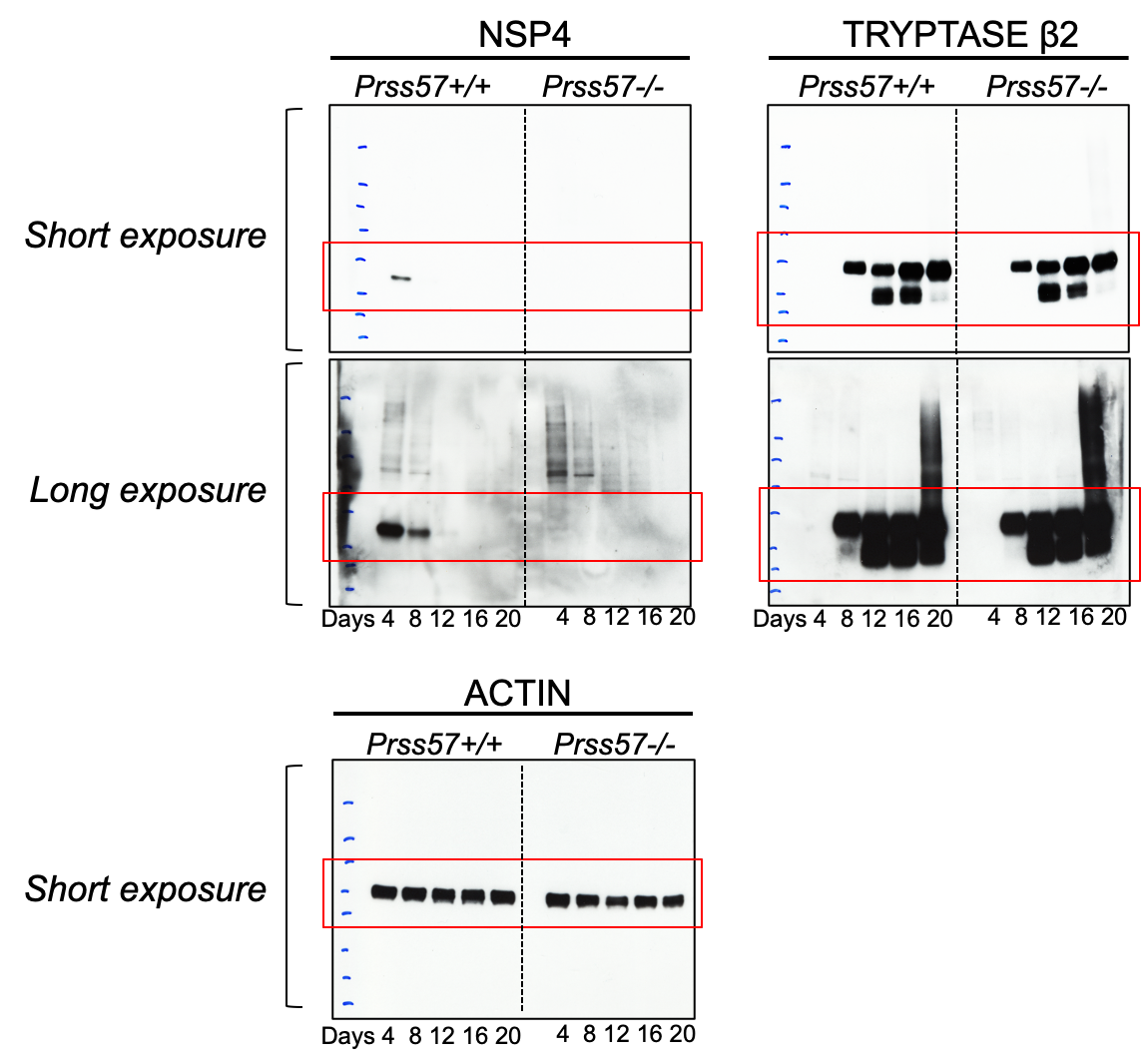


Supplementary Figure 1b. Western blot for NSP4 expression in total bone marrow.

NSP4

ACTIN

Supplementary Figure 3b. Western blot for NSP4 expression in bone marrow-resident neutrophils.


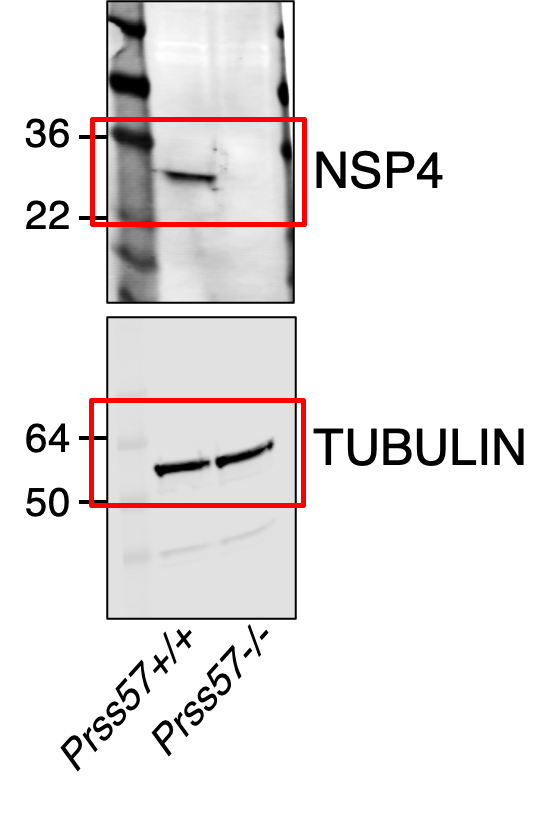


Supplementary Figure 4c. Western blot for various proteins expressed in primary GMPs.


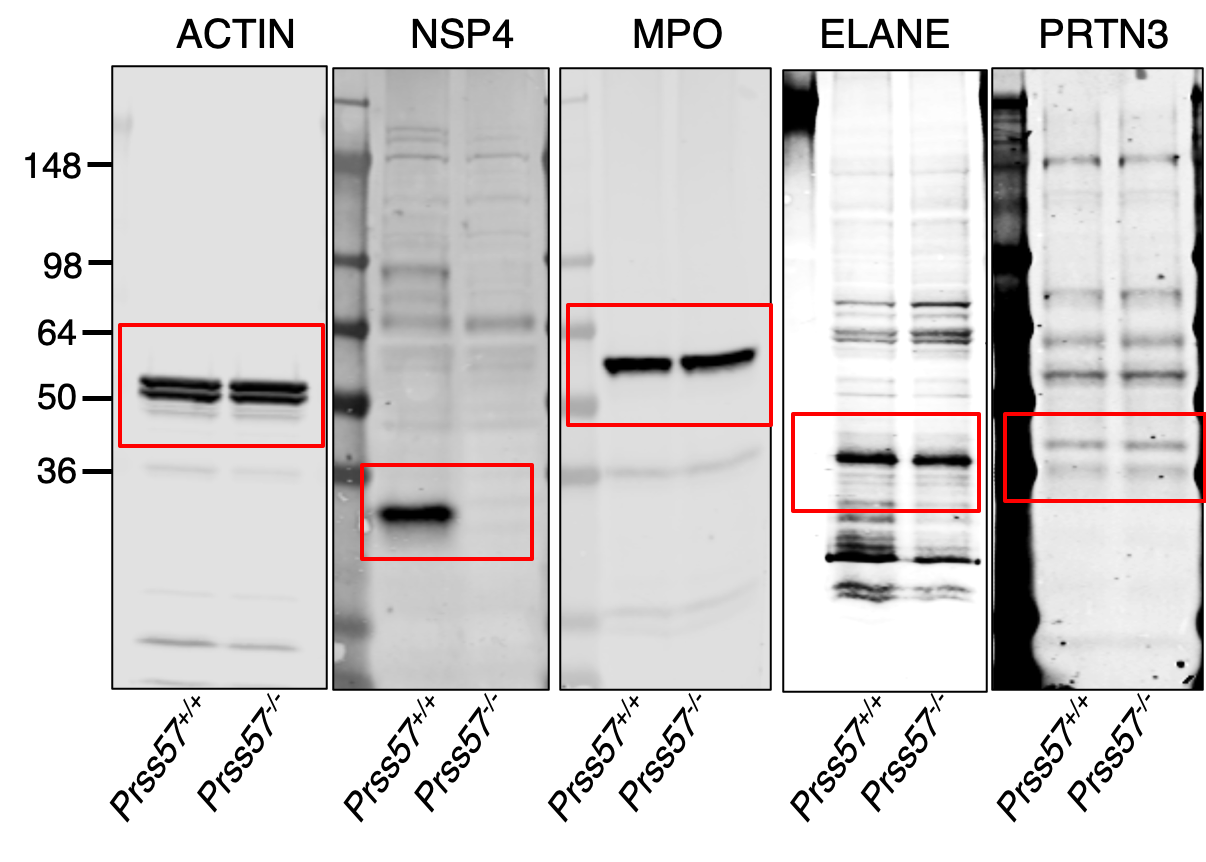


Supplementary Figure 4d. Western blot for various proteins expressed in primary GMPs


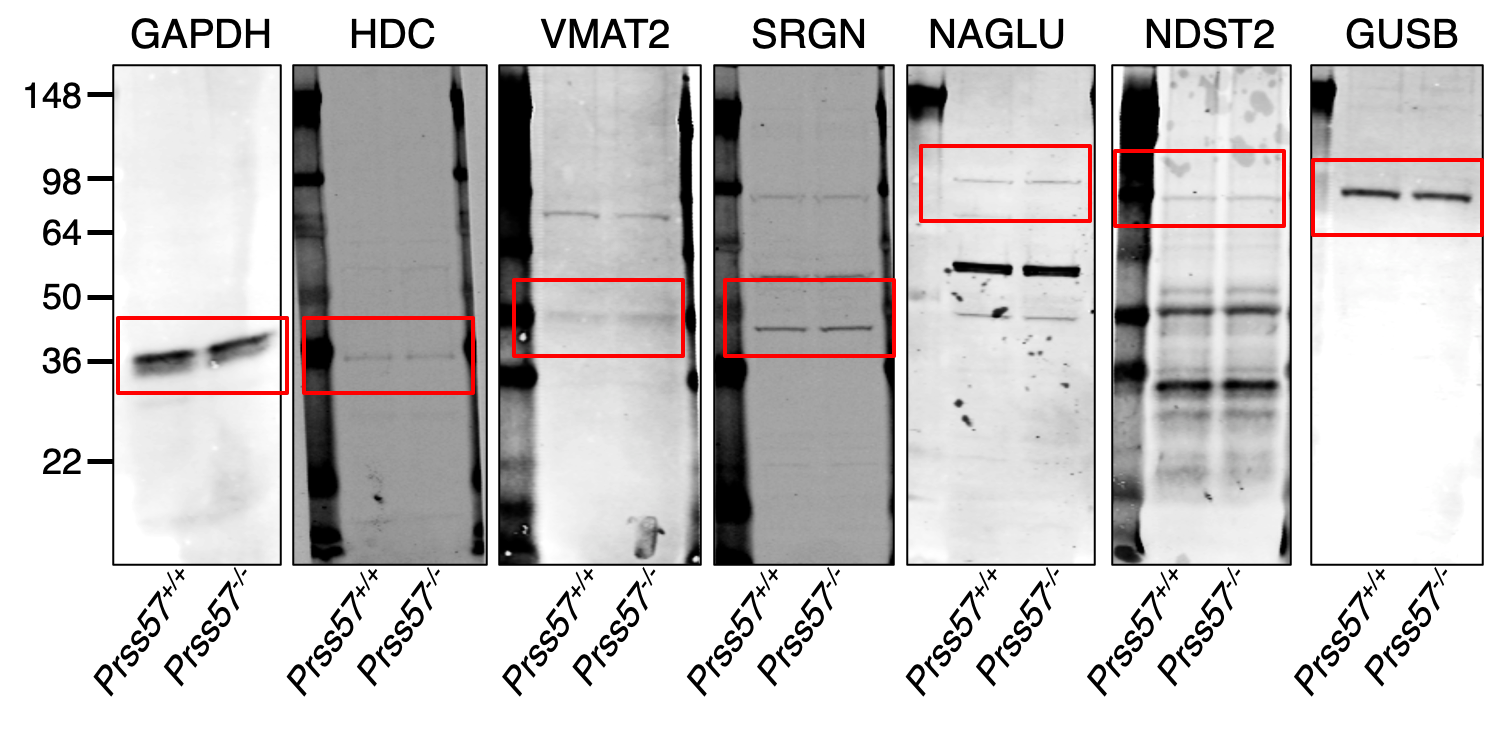


Supplementary Figure 5d. Western blot for neutrophil serine proteases expressed in bone marrow-resident neutrophils


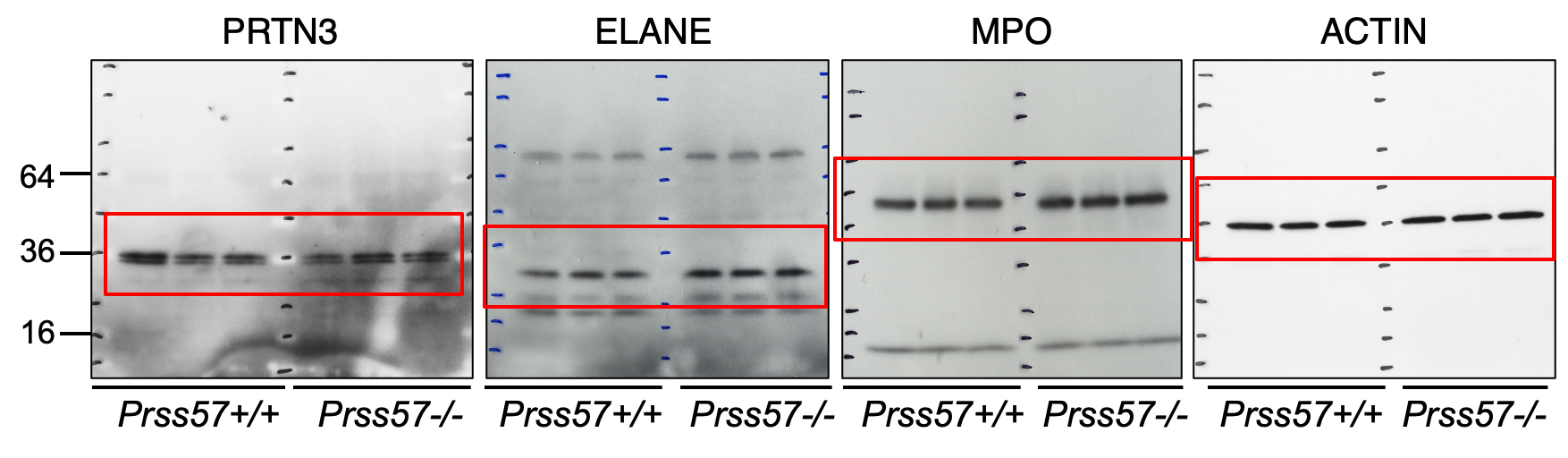


Supplementary Figure 9b. Western blot for mast cell proteases expressed in BMMCs


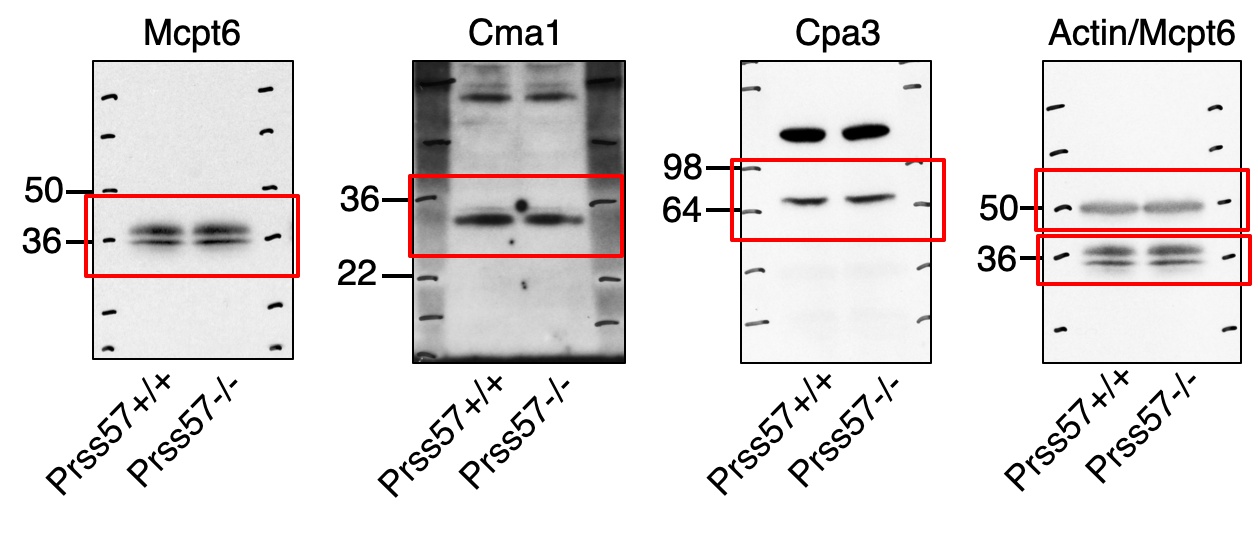


Supplementary Figure 9d. Western blot for mast cell proteins expressed in primary peritoneal mast cells


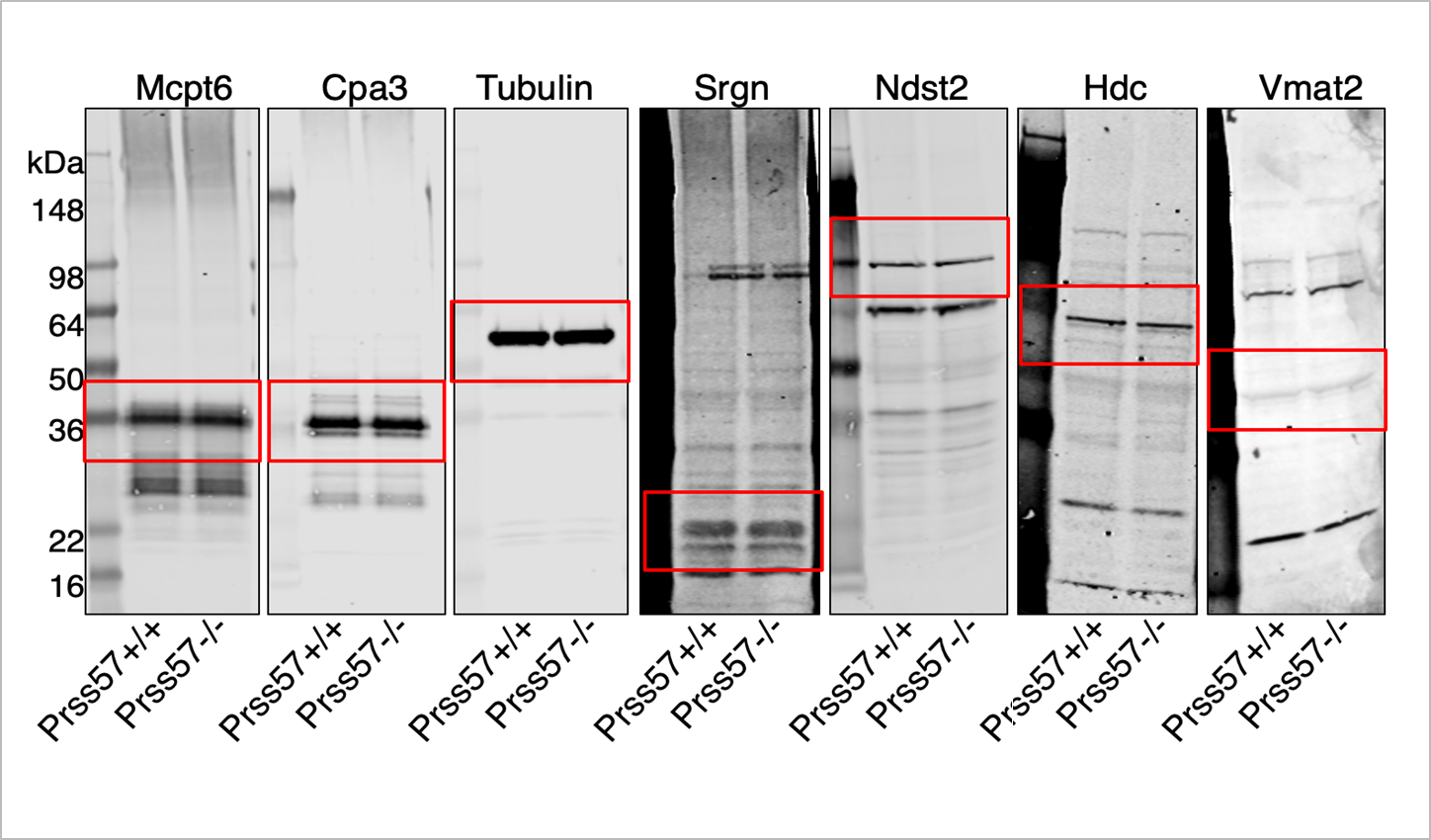

Supplement: Supplementary file 1 — Supplementary Information [file 42003_2020_1407_MOESM1_ESM.docx]
