# Supplementary material for: Neutrophil serine protease 4 is required for mast cell-dependent vascular leakage
Source: Commun Biol. 2020 Nov 19;3:687. doi: 10.1038/s42003-020-01407-0 (PMC7677402; doi:10.1038/s42003-020-01407-0)
Supplement: Supplementary file 2 — Description of Additional Supplementary Files [file 42003_2020_1407_MOESM2_ESM.docx]

Description of Additional Supplementary Files

Supplementary Data 1: The source data underlying Figs. 1-7 of the main manuscript and Supplementary Fig. 1-11
