# Supplementary material for: Neutrophil serine protease 4 is required for mast cell-dependent vascular leakage
Source: Commun Biol. 2020 Nov 19;3:687. doi: 10.1038/s42003-020-01407-0 (PMC7677402; doi:10.1038/s42003-020-01407-0)
Supplement: Supplementary file 4 — Reporting Summary [file 42003_2020_1407_MOESM4_ESM.pdf]

nature research

Corresponding author(s): Menno van Lookeren Campagne, Daniel Kirchhofer

Last updated by author(s): Sep 2, 2020

Reporting Summary

Nature Research wishes to improve the reproducibility of the work that we publish. This form provides structure for consistency and transparency in reporting. For further information on Nature Research policies, see Authors & Referees and the Editorial Policy Checklist .

Statistics

For all statistical analyses, confirm that the following items are present in the figure legend, table legend, main text, or Methods section.

n/a	Confirmed	
☐	☒	The exact sample size (*n*) for each experimental group/condition, given as a discrete number and unit of measurement
☐	☒	A statement on whether measurements were taken from distinct samples or whether the same sample was measured repeatedly
☐	☒	The statistical test(s) used AND whether they are one- or two-sided<br>*Only common tests should be described solely by name; describe more complex techniques in the Methods section.*
☐	☒	A description of all covariates tested
☐	☒	A description of any assumptions or corrections, such as tests of normality and adjustment for multiple comparisons
☐	☒	A full description of the statistical parameters including central tendency (e.g. means) or other basic estimates (e.g. regression coefficient) AND variation (e.g. standard deviation) or associated estimates of uncertainty (e.g. confidence intervals)
☐	☒	For null hypothesis testing, the test statistic (e.g. *F*, *t*, *r*) with confidence intervals, effect sizes, degrees of freedom and *P* value noted<br>*Give P values as exact values whenever suitable.*
☒	☐	For Bayesian analysis, information on the choice of priors and Markov chain Monte Carlo settings
☐	☒	For hierarchical and complex designs, identification of the appropriate level for tests and full reporting of outcomes
☐	☒	Estimates of effect sizes (e.g. Cohen's *d*, Pearson's *r*), indicating how they were calculated

Our web collection on statistics for biologists contains articles on many of the points above.

Software and code

Policy information about availability of computer code

Data collection	No software was used.
Data analysis	No software was used.

For manuscripts utilizing custom algorithms or software that are central to the research but not yet described in published literature, software must be made available to editors/reviewers. We strongly encourage code deposition in a community repository (e.g. GitHub). See the Nature Research guidelines for submitting code & software for further information.

Data

Policy information about availability of data

All manuscripts must include a data availability statement. This statement should provide the following information, where applicable:

- Accession codes, unique identifiers, or web links for publicly available datasets
- A list of figures that have associated raw data
- A description of any restrictions on data availability

Authors can confirm that all relevant data, including extended data files and source data files, are included in the manuscript. All data are available from the corresponding authors on reasonable request. All reagents are available from D.K. or M.V.L.C. under a material transfer agreement with Genentech Inc. The RNAseq datasets has been deposited in NCBI (identifier GSE138697) and will be accessible to the public once the paper is accepted. The source data underlying Figs. 1-7 of the main manuscript and Supplemental Figures 1-11 are provided as a source data file.

Field-specific reporting

Please select the one below that is the best fit for your research. If you are not sure, read the appropriate sections before making your selection.

Life sciences study design

All studies must disclose on these points even when the disclosure is negative.

Sample size	We chose the sample size based on the literature in the field and based on historical data from our lab. The sample size (n) for all animal studies is defined in the figure legends.
Data exclusions	No data were excluded from the analyses.
Replication	All attempts at replication were successful. All experiments were repeated at least 3 times. Please see figure legends for more details.
Randomization	In all studies, animals were age and gender-matched. No randomization protocol was applied as this was not relevant.
Blinding	All of the K/BxN studies, including the adoptive transfer experiments, were blinded. These experiments are depicted in figures 6 and 7.

Reporting for specific materials, systems and methods

We require information from authors about some types of materials, experimental systems and methods used in many studies. Here, indicate whether each material, system or method listed is relevant to your study. If you are not sure if a list item applies to your research, read the appropriate section before selecting a response.

Materials & experimental systems

n/a	Involved in the study	
☐	☒	Antibodies
☒	☐	Eukaryotic cell lines
☒	☐	Palaeontology
☐	☒	Animals and other organisms
☒	☐	Human research participants
☒	☐	Clinical data

Methods

n/a	Involved in the study	
☒	☐	ChIP-seq
☐	☒	Flow cytometry
☒	☐	MRI-based neuroimaging

Antibodies

Antibodies used	All western blot antibodies used in the study are presented in Table S2. All flow cytometry and IHC antibodies are detailed in the methods sections (pages 12, 13, 14 and 16) pertaining to specific experiments.
Validation	All antibodies were validated by their manufacturer prior to purchasing. The catalog numbers are also provided in order to access more information regarding antibody source and validation profile.

Animals and other organisms

Policy information about studies involving animals; ARRIVE guidelines recommended for reporting animal research

Laboratory animals	6-8 week old C57BL/6N mice were used for all in vivo experiments . Female or male mice were used as indicated in the Methods section. Mice were obtained either from Genentech's breeding colonies or obtained from the Jackson Laboratories, Bar Harbor.
Wild animals	n/a
Field-collected samples	n/a
Ethics oversight	The Genentech institutional animal care and use committee (IACUC) responsible for ethical compliance approved all animal protocols.

Note that full information on the approval of the study protocol must also be provided in the manuscript.

Flow Cytometry

Plots

Confirm that:

- [x] The axis labels state the marker and fluorochrome used (e.g. CD4-FITC).
- [x] The axis scales are clearly visible. Include numbers along axes only for bottom left plot of group (a 'group' is an analysis of identical markers).
- [x] All plots are contour plots with outliers or pseudocolor plots.
- [x] A numerical value for number of cells or percentage (with statistics) is provided.

Methodology

Sample preparation	Please see Methods section (pages 12-13) for the complete details of 1) tissue isolation, 2) generation of single cell suspension, 3) staining procedure for extracellular and intracellular stainings, 4) list of antibodies and their respective fluorophores, and 5) downstream assays.<br><br>Please see Methods section (Page 16) for details of adoptive transfer study and sample characterization by flow cytometry.
Instrument	The Aria is a Becton Dickinson FACSAria Fusion with 355nm, 405nm, 488nm, 561nm, and 638nm lasers.<br>The Symphony is a Becton Dickinson Dual Symphony A5 with the same 5 lasers.
Software	BD FACS DIVA
Cell population abundance	The purity (>95%) of FAC-sorted samples was determined by post-sort analysis of the sorted populations.
Gating strategy	Figure 1. Intracellular staining and flow cytometry analysis for NSP4 expression in bone marrow cells. Density plots describe the gating strategy and histogram overlays of Prss57+/+, Prss57-/-, and rat IgG2b isotype staining are shown for each gated population: 1) LSK: Lin-, Sca-1+, CD117+ cells; 2) Megakaryocyte/erythrocyte progenitors (MEP): Lin-, Sca-1-, CD117+, CD34-, CD16/32lo; 3) Common myeloid progenitors (CMP): Lin-, Sca-1-, CD117+, CD34+, CD16/32lo; 4) Granulocyte-macrophage progenitors (GMP): Lin-, Sca-1-, CD117+, CD34+, CD16/32hi.<br><br>Figure S3a. Representative FAC-sorting strategy for the isolation of bone marrow-resident neutrophils.<br><br>Figure S4a. Representative FAC-sorting strategy for the isolation of primary GMPs from the bone marrow (Lin-, Sca-1-, CD117+, CD34+, CD16/32hi).<br><br>Figure S6c. Representative flow cytometry plots of in vitro differentiated BMMCs and PCMCs after 4 weeks of differentiation and stained for surface expression of the mast cell receptors c-KIT and FceRIa.<br><br>Figure S9c. Representative FAC-sorting strategy for the isolation of peritoneal mast cells,<br><br>Figure S10. Flow cytometry analysis of FAC-sorted GMPs.<br><br>Figure S11. Representative flow cytometry plots of mast cells in adoptive transfer studies.

- [x] Tick this box to confirm that a figure exemplifying the gating strategy is provided in the Supplementary Information.
